# N-GSDMD trafficking to neutrophil organelles facilitates IL-1β release independently of plasma membrane pores and pyroptosis

**DOI:** 10.1038/s41467-020-16043-9

**Published:** 2020-05-05

**Authors:** Mausita Karmakar, Martin Minns, Elyse N. Greenberg, Jose Diaz-Aponte, Kersi Pestonjamasp, Jennifer L. Johnson, Joseph K. Rathkey, Derek W. Abbott, Kun Wang, Feng Shao, Sergio D. Catz, George R. Dubyak, Eric Pearlman

**Affiliations:** 1 Department of Physiology and Biophysics, Case Western Reserve University, Cleveland, OH USA; 2 Department of Physiology and Biophysics, and the Department of Ophthalmology, University of California, Irvine, CA USA; 3 The Scripps Research Institute, La Jolla, CA USA; 4 Department of Pathology, Case Western Reserve University, Cleveland, OH USA; 5 National Institute of Biological Sciences, Beijing, China

**Keywords:** Autophagy, Interleukins, Immune cell death, Inflammasome, Neutrophils

## Abstract

Gasdermin-D (GSDMD) in inflammasome-activated macrophages is cleaved by caspase-1 to generate N-GSDMD fragments. N-GSDMD then oligomerizes in the plasma membrane (PM) to form pores that increase membrane permeability, leading to pyroptosis and IL-1β release. In contrast, we report that although N-GSDMD is required for IL-1β secretion in NLRP3-activated human and murine neutrophils, N-GSDMD does not localize to the PM or increase PM permeability or pyroptosis. Instead, biochemical and microscopy studies reveal that N-GSDMD in neutrophils predominantly associates with azurophilic granules and LC3^+^ autophagosomes. N-GSDMD trafficking to azurophilic granules causes leakage of neutrophil elastase into the cytosol, resulting in secondary cleavage of GSDMD to an alternatively cleaved N-GSDMD product. Genetic analyses using ATG7-deficient cells indicate that neutrophils secrete IL-1β via an autophagy-dependent mechanism. These findings reveal fundamental differences in GSDMD trafficking between neutrophils and macrophages that underlie neutrophil-specific functions during inflammasome activation.

## Abstract (web summary)

In macrophages, IL-1β secretion is mediated by N-GSDMD pores in the plasma membrane (PM). Here the authors show that in neutrophils, IL-1β secretion occurs in the absence of PM pores, via autophagosomes; N-GSDMD does not traffic to PM but to azurophilic granules, thereby releasing neutrophil elastase which cleaves further N-GSDMD into alternative fragments.

## Introduction

The Gasdermin (GSDM) family of proteins are regulators of innate immune and cell death responses. Pyroptosis, a pro-inflammatory mode of lytic cell death mediated by Gasdermin D (GSDMD) is the best-characterized response^1,2^. GSDMs are expressed as ~50 kDa cytosolic pro-proteins with N-terminal effector and C-terminal regulatory domains, and a binding interface between the C-terminal domain and the ~30 kDa N-GSDM effector moiety maintains pro-GSDM in an auto-inhibited conformation. Disruption of this interface by proteolytic cleavage of linker loops or mutation of key residues induces conformational rearrangement of N-GSDM subunits (reviewed in refs. ^1,3,4^) to expose sites for interaction with anionic phospholipids on accessible leaflets of membrane bilayers. This facilitates N-GSDM oligomerization and drives insertion of multiple β-hairpins through the targeted bilayer to assemble macropores (10–18 nm inner diameters). Assembly of N-GSDM pores in the plasma membrane markedly increases its permeability to macromolecules (up to 20 kDa), metabolites, ions, and major osmolytes, resulting in rapid collapse of cellular integrity to facilitate pyroptosis^5–7^. In infected tissues, pyroptosis eliminates the replicative niche of intracellular bacteria within dying macrophages to entrap bacteria for ingestion by recruited neutrophils^8^.

Physiological roles for GSDMD in both pyroptosis and IL-1β release during inflammasome signaling have been extensively characterized in macrophages and other mononuclear leukocytes. IL-1β lacks the signal sequence required for conventional exocytotic secretion via the endoplasmic reticulum/Golgi pathway and is therefore released by non-classical export mechanisms^9^. During canonical inflammasome signaling, caspase-1 cleaves pro-IL-1β to the 17 kDa bioactive cytokine, and cleaves the 52 kDa pro-GSDMD to 31 kDa N-GSDMD products, which oligomerize at the macrophage plasma membrane to generate pores that function as direct conduits for IL-1β efflux and mediators of pyroptosis^10^. In murine macrophages, glycine markedly delays pyroptosis (via an unknown mechanism), but does not inhibit IL-1β release or assembly of N-GSDMD pores^11,12^.

Neutrophils, which are recruited in large numbers following infection or tissue damage, are also a major source of IL-1β. We and others reported that neutrophils release IL-1β in the absence of pyroptosis during canonical NLRP3 inflammasome activation^13–15^. Despite this independence from pyroptosis, two recent studies used *Gsdmd*^*−/−*^ mice to show that neutrophil IL-1β release is reduced in the absence of GSDMD, similar to macrophages^16,17^. Although the mechanism for the absence of GSDMD-mediated pyroptosis in neutrophils was not directly investigated, the authors suggested that the non-lytic IL-1β release reflects direct efflux via plasma membrane N-GSDMD pores as with macrophages^12^, and may be coupled with a robust ability of neutrophils to remove N-GSDMD pores from the plasma membrane via membrane repair, as also described for macrophages^18^. However, accumulation of functional N-GSDMD pores in the neutrophil plasma membrane or roles for membrane repair in limiting pore numbers in neutrophils have not been explicitly evaluated.

In the current study, we describe an alternative mechanism for the resistance of inflammasome-activated neutrophils to pyroptosis despite generation of pore-competent N-GSDMD products. Using functional analyses of plasma membrane permeability, biochemical analyses of subcellular fractions, and super-resolution imaging of single neutrophils with a novel monoclonal antibody that recognizes N-GSDMD but not pro-GSDMD, we find that unlike macrophages, inflammasome-activated neutrophils: (a) do not accumulate functional N-GSDMD pores in the plasma membrane; (b) do not activate Ca^2+^-regulated plasma membrane repair; (c) do not traffic N-GSDMD protein to the plasma membrane, instead trafficking N-GSDMD to azurophilic (primary) granules and autophagosomes; and (d) release IL-1β via an autophagy machinery-dependent pathway. Further, N-GSDMD permeabilization of azurophilic granules releases neutrophil elastase into the cytosol, which mediates a secondary cascade of serine protease–dependent GSDMD processing. These results demonstrate that dynamic distribution of N-GSDMD can involve binding to membranes of abundant intracellular organelles, in addition to the plasma membrane, to provide neutrophil-specific pathways of GSDMD function in innate immunity.

## Results

### Absence of plasma membrane GSDMD pores in neutrophils

**Fig. 1:**
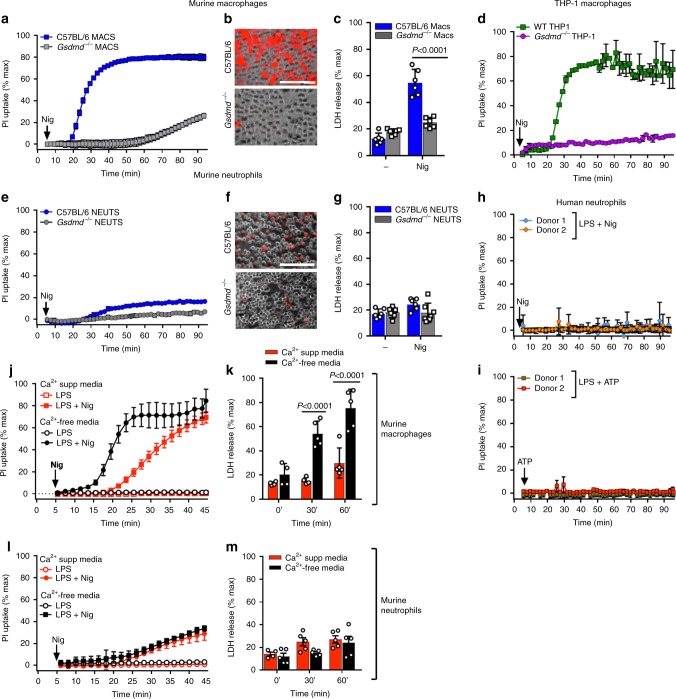
Plasma membrane permeability and Ca^2+^—dependent plasma membrane repair in NLRP3-activated human and murine macrophages and neutrophils. **a**–**c** NLRP3-activated (LPS/nigericin) bone marrow derived macrophages from C57BL/6 and *Gsdmd* ^−/−^ mice showing a time course of propidium iodide (PI) uptake (**a**), representative PI positive cells after 45 min (**b**), and LDH release after 90 min (**c**). 5 mM glycine was added to the extracellular media for PI imaging of C57BL/6 and *Gsdmd*^−/−^ macrophages during nigericin stimulation to avoid cell lysis but was not added to neutrophil cultures. **d** Time course of PI uptake by LPS/nigericin activated WT and *Gsdmd*^−/−^ THP-1 human macrophages. **e**–**g** NLRP3-activated bone marrow neutrophils from C57BL/6 and *Gsdmd* ^−/−^ mice showing PI uptake (**e**, **f**) and LDH release after 90 min incubation (**g**). **h**, **i** PI uptake by LPS/nigericin and LPS/ATP—activated peripheral blood neutrophils from two healthy human donors. (IL-1β production is shown in Supplemental Fig. 3). **j**–**m** PI uptake and LDH release (45 min) by murine macrophages (**j**, **k**) and neutrophils (**l**, **m**) stimulated with nigericin in either Ca^2+^ supplemented or Ca^2+^ free media. Time course of PI uptake (**a**, **d**, **g**, **h**, **i**, **j**, **l**) are mean ± SD of 4 independent experiments (*n* = 4); LDH data (**c**, **g**, **k**, **m**) are mean ± SD of biological replicates for each condition from 5 or more independent experiments (*n* = 5 data points). *p-*Values were derived by two-way ANOVA using Sidak’s multiple comparisons test; *p* < 0.05 is significant, *n.s*.: not significant. Panels B, F: scale bar = 100 µm. Source data for panels **a**, **c**, **d**, **e**, **g**, **h**, **i**, **j**, **k**, **l**, **m** are provided in the separate Source Data file.

Maximal IL-1β release by neutrophils requires GSDMD as recently reported^16,17^ and confirmed by our data (Supplementary Fig. 1). However, no studies have directly examined if N-GSDMD forms pores in the neutrophil plasma membrane, following activation of NLRP3 inflammasomes by nigericin or ATP. We found that as reported, nigericin triggered robust propidium iodide (PI) influx in C57BL/6 but not *Gsdmd*^*−/−*^ macrophages (Fig. 1a, b). Imaging of activated macrophages was performed in the presence of glycine to inhibit pyroptosis. However, in the absence of glycine, nigericin stimulated LDH release from C57BL/6, but not *Gsdmd*^*−/−*^ macrophages (Fig. 1c). ATP triggered similar PI influx and LDH release responses that were GSDMD-dependent (Supplementary Fig. 2a–c). We also observed rapid PI uptake in nigericin-stimulated human THP-1 macrophages, but not in CRISPR generated *Gsdmd*^−/−^ THP-1 cells (Fig. 1d).

In marked contrast to macrophages, there was no increase in PI uptake or LDH release in bone marrow neutrophils from C57BL/6 mice following stimulation with nigericin or ATP (Fig. 1e–g, Supplementary Fig. 2d–f). These differences in PI uptake between macrophages and neutrophils were confirmed by quantitative flow cytometry (Supplementary Fig. 2g–j). Similarly, neither PI uptake (Fig. 1h, i) nor LDH release (Supplementary Fig. 3a) were detected in LPS-primed blood neutrophils from healthy human donors (*n* = 8) stimulated with nigericin or ATP, even though these stimuli induced robust IL-1β secretion (Supplementary Fig. 3b). Thus, murine and human neutrophils do not accumulate functional GSDMD pores in the plasma membrane at time points corresponding to high rates of IL-1β release. The small increase in PI accumulation by nigericin-treated C57BL/6 neutrophils relative to *Gsdmd*^*−/−*^ neutrophils (Fig. 1e) likely reflects heterogeneity among the immature and mature neutrophil subpopulations in bone marrow and was not observed in stimulated human blood neutrophils (Fig. 1h).

Robust Ca^2+^ influx-dependent membrane repair mechanisms are activated in response to accumulation of GSDMD pores in the plasma membrane of macrophages to counteract pyroptotic lysis^18^. We compared the PI influx and LDH release responses in murine neutrophils versus macrophages stimulated with nigericin either in Ca^2+^-free or Ca^2+^-supplemented media. As shown in Fig. 1j, k, the absence of extracellular Ca^2+^ (and consequent Ca^2+^ influx) markedly increased both PI influx and LDH release in NLRP3-activated macrophages, which correlated with enhanced IL-1β release (Supplementary Fig. 4a). In contrast, the absence of extracellular Ca^2+^ did not facilitate or alter PI permeability, LDH release or IL-1β secretion in NLRP3-activated neutrophils (Fig. 1l, m, Supplementary Fig. 4b).

The cryo-EM structures of GSDMD and GSDMA3 membrane pores indicate similar topology to members of the MACPF/CDC (Membrane Attack Complex Perforin-like/ Cholesterol Dependent Cytolysin) family of pore-forming proteins^19^. We therefore examined if the absence of GSDMD pore formation in neutrophils was due to intrinsic resistance of their plasma membranes to the actions of pore-forming proteins. C57BL/6 and *Gsdmd*^*−/−*^ neutrophils were stimulated with sub-lytic concentrations of the *Streptococcus pneumoniae* exotoxin pneumolysin (Ply), which is a MACPF/CDC protein. Ply induced robust PI influx in both neutrophils and macrophages, although C57BL/6 macrophages exhibited greater PI influx compared to *Gsdmd*^*−/−*^ macrophages in response to Ply (Supplementary Fig. 5a). We used the NLRP3 inhibitor MCC950 to show that this was due to the combined actions of primary influx via Ply pores plus secondary influx via N-GSDMD pores, which accumulate as a consequence of Ply pore-mediated K^+^ efflux and NLRP3/caspase-1 inflammasome activation (Supplementary Fig. 5b).

Thus, during NLRP3 inflammasome activation in neutrophils, GSDMD does not form pores in plasma membranes and this is not a consequence of Ca^2+^-dependent membrane repair or intrinsic resistance to MACPF/CDC-like pore-forming proteins.

### N-GSDMD does not localize to neutrophil plasma membranes

**Fig. 2:**
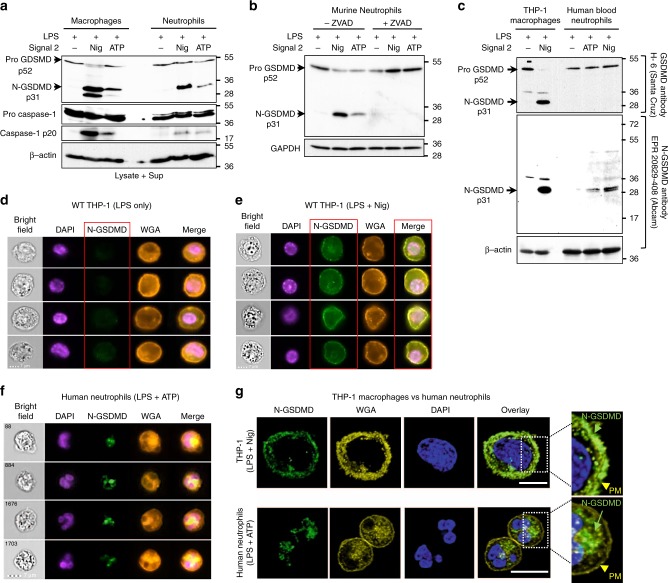
Subcellular localization of N-GSDMD in neutrophils versus macrophages. **a** GSDMD western blot (Abcam EPR 19828) from total cell lysate + supernatant of LPS-primed, NLRP3-stimulated (45 mins) bone marrow derived macrophages and bone marrow neutrophils from C57BL/6 mice (neutrophil and macrophage lysates were run side by side on the same gels). All neutrophil lysates were generated in the presence of 5 mM DFP (serine protease inhibitor) in addition to a standard protease inhibitor cocktail. **b** LPS-primed neutrophils incubated with the pan-caspase inhibitor zVAD (1.5 µM) for 30 min before adding nigericin or ATP. **c** LPS - primed THP-1 macrophages (PMA-differentiated) and human donor neutrophils were stimulated 45 min with 10 μM nigericin or 3 mM ATP. Western blots of total cell lysates from THP-1 macrophages (100 μg) and human neutrophils (200 μg) were probed with either Santa Cruz clone H-6 that recognizes pro- and cleaved GSDMD, or with EPR 20829-408 (Abcam) that specifically recognizes p31 N-GSDMD. β-actin was the loading control. Molecular weight markers in kDa are shown on the right. **d**–**g** NLRP3-activated THP-1 human macrophages and human neutrophils immunostained with the N-GSDMD specific antibody (Abcam EPR 20829-408). **d**, **e** Representative Imagestream images of WT THP-1 cells stimulated with LPS only (no GSDMD cleavage) (**d**) or with LPS/nigericin of WT THP-1 macrophages (**e**) and co-stained with wheat germ agglutinin (WGA) to identify the plasma membrane and N-GSDMD colocalization. (Supplemental Fig. 7 shows Imagestream of *Gsdmd*^*−/−*^ THP-1 macrophages stimulated with LPS/nigericin.) **f**, **g** WGA and N-GSDMD staining in NLRP3-activated (LPS + ATP) peripheral blood neutrophils captured by Imagestream (**f**) or confocal microscopy (Huygens deconvolution) images (**g**). Yellow and green arrows in the enlarged images show plasma membrane (PM) and N-GSDMD, respectively. Western blots are representative of 3 independent experiments (*n* = 3), and Imagestream and confocal microscopy images are representative of neutrophils from three donors (*n* = 3). Source data for all western blots in Panels A, B, C are provided as uncropped blots in Supplementary Information, Supplementary Fig. 15.

To identify mechanisms underlying the absence of plasma membrane GSDMD pores in neutrophils, we examined GSDMD processing and localization of N-GSDMD by western blot and immunofluorescence imaging. Neutrophil extracts were routinely prepared in RIPA lysis buffer supplemented with diisopropyl fluorophosphate (DFP), an irreversible inhibitor of the multiple serine proteases that are present at high levels in neutrophil granules. By combining whole cell lysates and extracellular supernatants for western blot analysis, we observed that the processing of p52 pro-GSDMD to p31 N-GSDMD in NLRP3-activated murine neutrophils was qualitatively similar to murine macrophages (Fig. 2a). However, the quantity of accumulated p31 N-GSDMD in neutrophils was lower than in macrophages. Using two different antibody clones that target murine GSDMD, we found that p52 pro-GSDMD levels were similar in LPS-primed neutrophils and macrophages prior to NLRP3 inflammasome activation (Fig. 2a and Supplementary Fig. 6a); however, there was less p31 N-GSDMD in NLRP3-activated neutrophils relative to macrophages, which correlated with lower production of cleaved caspase-1 (Fig. 2a). Pro-GSDMD cleavage to the p31 N-GSDMD in neutrophils was blocked by the pan-caspase inhibitor zVAD (Fig. 2b), indicating that as with macrophages^3,20^, accumulation of neutrophil p31 N-GSDMD was dependent on caspases.

Nigericin-stimulated murine macrophages resulted in a 28 kDa N-GSDMD product in addition to the p31 N-GSDMD (Fig. 2a and Supplementary Fig. 6b). Accumulation of this smaller GSDMD product was suppressed by DEVD-fmk (Supplementary Fig. 6b), indicating a role for caspase-3/7 in addition to caspase-1 in GSDMD cleavage. This is consistent with reports that caspase-1 secondarily activates caspase-7 in inflammasome-stimulated macrophages^21^. Although caspase-3/7 can cleave human and murine GSDMD at Asp-87^22^, murine, but not human, GSDMD also contains Asp-27 within an IPVD motif. Combined cleavage of murine GSDMD at Asp-276 by caspase-1 and Asp-27 by caspase-7 would generate a 27.4 kDa product as shown.

We also compared GSDMD expression and processing in human blood neutrophils and human THP-1 macrophages using two different antibodies for human GSDMD (Fig. 2c). The H-6 rabbit monoclonal antibody (Santa Cruz) recognizes both pro-GSDMD and p31 N-GSDMD, whereas a novel rabbit monoclonal antibody, initially generated by Shao and colleagues but now commercially available (Abcam EPR20829-408), recognizes the human p31 N-GSDMD cleavage product, but not pro-GSDMD. We found that pro-GSDMD in THP-1 cells and human neutrophils was detected by H-6 Ab, but not by EPR20829-408 (Fig. 2c), which detected p31-N-GSDMD in both neutrophils and THP-1 cells. Although stimulation of THP-1 macrophages with nigericin for 45 min resulted in near-complete processing of pro-GSDMD as detected by the H6 Ab, the p31-N-GSDMD in ATP- or nigericin-stimulated human neutrophils was below the H6 detection threshold. Thus, human neutrophils, like murine neutrophils, accumulate p31 N-GSDMD during NLRP3 inflammasome signaling but at quantitatively lower levels than macrophages.

The absence of functional plasma membrane pores in neutrophils suggested that pore-competent p31 N-GSDMD products do not efficiently traffic to the plasma membrane. We examined localization of N-GSDMD using the N-GSDMD-selective EPR208209 Ab by single cell imaging (Imagestream) and confocal microscopy. N-GSDMD co-localized with the plasma membrane marker wheat germ agglutinin (WGA) in LPS/nigericin-activated THP-1 macrophages, whereas THP-1 cells primed with LPS alone lacked anti-N-GSDMD reactivity (Fig. 2d, e). There was also no N-GSDMD staining in NLRP3-stimulated *Gsdmd*^−/−^ THP-1 macrophages (Supplementary Fig. 7). In contrast to the THP-1 macrophages, N-GSDMD was not detected in the plasma membrane of NLRP3-activated human neutrophils by Imagestream analyses, but rather accumulated within intracellular loci (Fig. 2f). Confocal microscopy revealed discrete N-GSDMD punctate staining in the cytoplasm of neutrophils, whereas N-GSDMD localized to the cell surface in macrophages (Fig. 2g).

Therefore, even though p31 N-GSDMD is generated in caspase-1-activated human neutrophils, it does not localize to the plasma membrane and consequently does not form functional cell surface pores. Rather, the N-GSDMD accumulates as intracellular puncta that are indicative of organelles.

### N-GSDMD mediates cytosolic release of neutrophil elastase

**Fig. 3:**
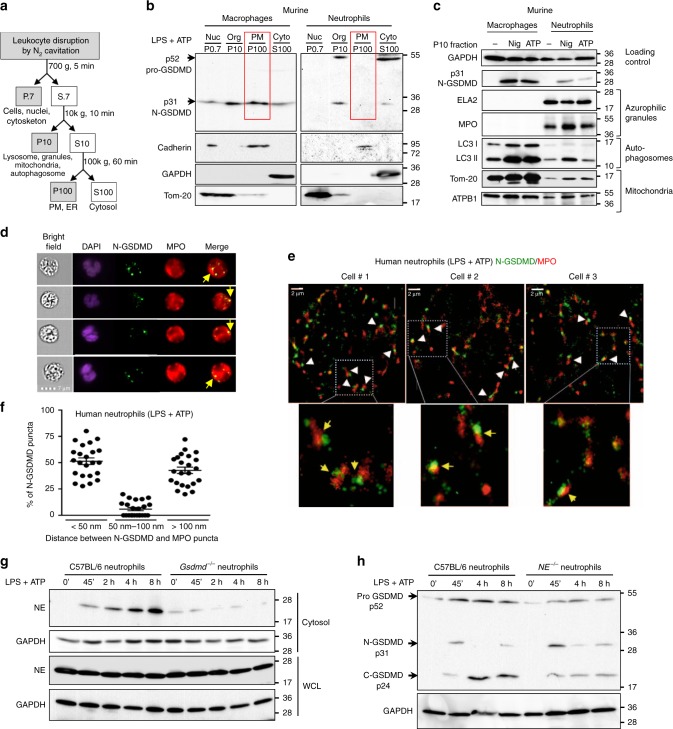
Localization of neutrophil N-GSDMD to azurophilic granules and release of active neutrophil elastase to the cytosol. **a** Schematic of subcellular fractionation of organelles, plasma membrane (PM) and cytosolic fractions. **b** Subcellular fractionation of cell homogenate of LPS + ATP-stimulated neutrophils and macrophages. **P0.7 (nuclei, undisrupted cells):** pellet from 0.7k *g*; **P10 (organelle fraction)**: pellet from 10k *g*; **P100 (plasma membrane fraction)**: pellet from 100k *g*; and **S100 (cytosolic fraction):** supernatant from 100k *g*. Tom 20, Cadherin and GAPDH are markers for mitochondria, PM and cytosolic fractions, respectively. **c** P10 organelle fractions of NLRP3-activated C57BL/6 macrophages and neutrophils. Neutrophil elastase (ELA2), and myeloperoxidase (MPO) indicate azurophilic granules; LC3I and LC3II are markers for autophagosomes; Tom-20 and ATBP1 are mitochondrial markers, and GAPDH was the loading control. **d**, **e** LPS + ATP stimulated human neutrophils immunostained with antibodies to N-GSDMD (EPR 20829-408) and MPO. Representative images were acquired by Imagestream (**d**) or by super-resolution microscopy (**e**). Arrows show overlap of N-GSDMD (green) and MPO (red) (**d**), and proximity is indicated by arrowheads of N-GSDMD (green) and MPO (red) in three representative neutrophils, and by arrows in enlarged images (lower panels) of super resolution microscopy images (**e**). Scale bar = 2 µm. Quantification of N-GSDMD proximity to MPO (**f**); each data point represents one neutrophil (median of 12 foci measured per cell), and percent of puncta where N-GSDMD is <50 nm, 50–100 nm, or >100 nm from MPO. **g, h** Time course of NE release into cytosol, and secondary GSDMD processing by cytosolic NE in LPS/ATP activated bone marrow neutrophils. Neutrophil elastase in cytosolic fractions and whole cell lysates of C57BL/6 and *Gsdmd*^−/−^ neutrophils (**g**), and GSDMD in whole cell lysates of neutrophils from C57BL/6 and neutrophil elastase-deficient *(NE*^−/−^*)* mice (probed with EPR19828) (**h**). All neutrophil lysates were generated in the presence of DFP and standard protease inhibitors. Western blots are representative of at least 3 independent experiments (*n* = 3). Molecular weight markers in KDa are indicated to the right. Images are representative of four different human donor neutrophils (*n* = 4). Source data for all western blots in Panels **b**, **c**, **g**, **h** are provided as uncropped blots in Supplementary Information, Supplementary Figs. 16 and 17. Source data for panel **f** is provided in the separate Source Data file.

We used biochemical and imaging approaches to identify subcellular N-GSDMD localization in NLRP3-activated murine neutrophils compared with macrophages. Detergent-free cell homogenates were generated by N_2_ cavitation, and were serially centrifuged as outlined in Fig. 3a: (a) 700 *g* to remove nuclei and undisrupted cells, (b) 10k *g* to yield P10 subcellular organelle fractions, and (c) 100k *g* to separate plasma membranes (P100) from the cytosol (S100). Proteins from each fraction were resolved by SDS-PAGE, and pro- and cleaved forms of GSDMD were detected by western blot. Consistent with previous analyses (reviewed in^3,20^), p31-N-GSDMD accumulated in the P100 fraction of LPS/ATP-stimulated macrophages, which was enriched in the plasma membrane marker cadherin (Fig. 3b), and also in the P10 fraction. The major contrast with macrophages was that the p31-N-GSDMD was not detected in the P100 fraction of LPS/ATP-activated neutrophils, although as with macrophages, N-GSDMD was detected in the S100 cytosol and P10 granule fractions (Fig. 3b). Similar results were observed in subcellular fractions prepared from LPS/nigericin-stimulated neutrophils and macrophages (Supplementary Fig. 8a). Neutrophils primed with LPS but not inflammasome-activated showed only full-length pro-GSDMD in the cytosol (Supplementary Fig. 8b). The stimulated neutrophils used in these fractionations released mature IL-1β and caspase-1 p20 into the extracellular media despite the absence of p31 N-GSDMD in the plasma membrane (Supplementary Fig. 8c). Moreover, p31 N-GSDMD was released into the culture supernatants of stimulated macrophages, but not neutrophils (Supplementary Fig. 8d), consistent with pyroptosis of macrophages, but not neutrophils.

The presence of p31 N-GSDMD in P10 fractions from NLRP3-activated neutrophils and macrophages, but not unstimulated cells, correlated with the presence of multiple intracellular organelle markers in both cell types; these included mitochondria (Tom-20 and ATBP1) and autophagosomes (LC3-II) (Fig. 3c). However, the P10 fraction of neutrophils, but not macrophages, was enriched in azurophilic granule markers (myeloperoxidase/MPO and neutrophil elastase/NE). To determine if N-GSDMD localizes to azurophilic granules, LPS-primed human blood neutrophils were stimulated for 45 min with ATP prior to immunostaining with the N-GSDMD-selective EPR208209 antibody and anti-MPO. Cells were examined by Imagestream analysis (Fig. 3d) and by super-resolution imaging (Fig. 3e, f) using stochastic optical reconstruction microscopy (STORM). Representative cells detected by Imagestream analyses revealed punctate staining of N-GSDMD and MPO (Fig. 3d); however, this association was more apparent in super-resolution images, which revealed close proximity of N-GSDMD to MPO (<50 nm) in 25–75% total N-GSDMD puncta (Fig. 3e, f).

To ascertain if N-GSDMD association with azurophilic granules resulted in increased granule permeability as a consequence of pore formation in granule membranes, we fractionated bone marrow neutrophils from C57BL/6 and *Gsdmd*^*−/−*^ mice following LPS/ATP stimulation, isolated the organelle-free, S100 cytosolic fraction, and assayed for neutrophil elastase (NE) by western blot. We found that NE accumulated over time in the cytosol of C57BL/6 neutrophils, but not *Gsdmd*^*−/−*^ neutrophils (Fig. 3g), which is consistent with N-GSDMD forming pores in the membrane of primary granules to release NE into the cytosol.

NE recognizes V251 upstream of the D276 caspase-1 cleavage site in murine GSDMD to generate a pore-forming 28kD N-GSDMD product and a p24 C-GSDMD fragment^23^, which we also detected (Supplementary Fig. 9). These data also highlight the importance of including the DFP serine protease to suppress post-lysis GSDMD processing in neutrophil lysates. In contrast, there was no effect of DFP on GSDMD processing in macrophages which do not produce elastase (Supplementary Fig. 10). Given that the EPR19828 mAb detects the NE-generated p24 C-GSDMD and caspase-1 generated p31-N-GSDMD, we examined if cytosolic NE, released from p31-N-GSDMD-permeabilized granules, can mediate a secondary phase of GSDMD processing in neutrophils during sustained NLRP3 activation. LPS-primed C57BL/6 neutrophils were stimulated with ATP for up to 8 h, and caspase-1 and NE cleavage products were examined by western blot.

Caspase-1-generated p31 N-GSDMD rapidly accumulated during the initial 45 min of ATP stimulation, whereas NE-generated p24 C-GSDMD was detected at 4 h and 8 h after stimulation and was markedly reduced in elastase-deficient *NE*^*−/−*^ neutrophils (Fig. 3h). The residual p24 C-GSDMD that accumulated in *NE*^−/−^ neutrophils is likely due to other neutrophil serine proteases (PR3 and cathepsin G) released from azurophilic granules^23,24^. Moreover, the biphasic change in p31-N-GSDMD levels indicates that this initial caspase-1 cleavage product is further trimmed by the released serine protease(s) with consequent loss of the epitope recognized by the EPR19828 mAb. Despite the progressive accumulation of cytosolic elastase, prolonged ATP stimulation (8 h) did not increase plasma membrane permeability or induce pyroptotic LDH release, and secretion of IL-1β rapidly increased only during the initial 2 h (Supplementary Fig. 11a–c).

Collectively, these findings reveal distinct features of GSDMD signaling in NLRP3-activated neutrophils compared with macrophages that include: 1) a predominant trafficking of pore-forming p31-N-GSDMD products to azurophilic granules rather than the plasma membrane; and 2) a role for granule-derived serine proteases in mediating a secondary phase of GSDMD proteolytic cleavage following the caspase-1 cleavage initiated by inflammasome activation.

### N-GSDMD localizes to LC3+ autophagosomes in neutrophils

**Fig. 4:**
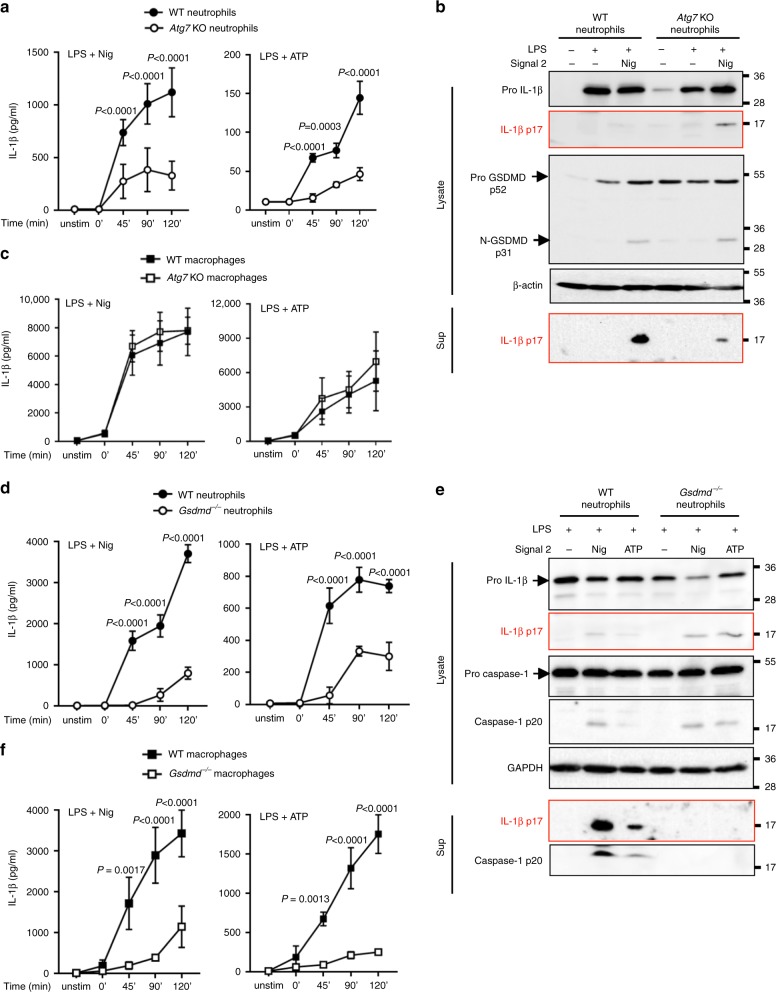
ATG7 and GSDMD - dependent IL-1β release from NLRP3 inflammasome - activated neutrophils or macrophages. **a**–**c** Bone marrow neutrophils and macrophages from *Atg7*^*f/f*^ (WT) and *Atg7*^*M∆*^ (*Atg7* KO) mice. **a** Time course of IL-1β secretion (**a**) and western blot of lysates and TCA precipitated supernatants of naïve (no LPS) or LPS primed neutrophils after stimulation with ATP or nigericin (**b**). **c** Time course of IL-1β secretion by bone marrow derived macrophages from *Atg7*^*f/f*^ (WT) and *Atg7*^*M∆*^ (*Atg7* KO) mice. **d**–**f** Bone marrow neutrophils and BM derived macrophages from C57BL/6 (WT) and *Gsdmd*^−/−^ mice showing IL-1β secretion from neutrophils (**d**), western blots of pro- and cleaved IL-1β and caspase-1 in cell lysates and supernatants (Sup; **e**) and IL-1β secretion by macrophages (**f**). For all experiments, cells were LPS primed for 3 h followed by 45 min stimulation with 10 µM nigericin or 3 mM ATP. Complete media + 2% FBS was used for all cell stimulations. Western blot data are representative of at least 3 biological repeats (*n* = 3); ELISA data are combined from 3 independent experiments (*n* = 3) with 4 replicates per condition. *p*-values were derived by two-way ANOVA and Sidak’s multiple comparisons test; *p* < 0.05: significant, n.s.: not significant. Source data for all western blots in Panels **b**, **e** are provided as uncropped blots in Supplementary Information, Supplementary Fig. 18. Source data for panels **a**, **c**, **d**, **e** are provided in the separate Source Data file.

Previous studies identified roles for autophagy proteins in the non-canonical release of IL-1β^25,26^. Our finding that N-GSDMD co-fractionates with LC3II^+^ autophagosomes (Fig. 3c) indicated that there may be a role for autophagy in GSDMD-dependent IL-1β secretion from neutrophils. We compared NLRP3 inflammasome-mediated production and release of IL-1β secretion in primary neutrophils from a control mouse strain expressing ATG7 in a floxed locus (*Atg7*^*f/f*^) versus neutrophils from mice with a myeloid-specific deletion of *Atg7* derived after crossing with LysMCre mice (*Atg7*^*M∆*^*)*^27^. Nigericin- or ATP-stimulated IL-1β release was significantly decreased in *Atg7*^*M∆*^ neutrophils relative to *Atg7*^*f/f*^ neutrophils (Fig. 4a), even though the cells were suspended in complete medium supplemented with amino acids to minimize autophagic activation secondary to mTOR suppression by amino acid starvation. Whereas cleaved IL-1β was mainly released into the extracellular supernatant from ATG7-expressing neutrophils, secretion of cleaved p17 IL-1β in *Atg7*^*M*∆^ neutrophils was lower, and correlated with increased intracellular retention (Fig. 4b). Similar levels of pro-IL-1β, pro-GSDMD and p31-N-GSDMD were detected in *Atg7*^*f/f*^ and *Atg7*^*M*∆^ neutrophil lysates (Fig. 4b). ASC oligomerization and caspase-1 processing were also identical in stimulated *Atg7*^*f/f*^ and *Atg7*^*M*∆^ neutrophils (Supplementary Fig. 12a). Thus, the role of ATG7 in IL-1β release is downstream of NLRP3 inflammasome assembly and caspase-1 cleavage of pro-IL-1β.

In contrast to neutrophils, there was no difference in IL-1β secretion between *Atg7*^*f/f*^ and *Atg7*^*M*∆^ macrophages during NLRP3 stimulation in complete amino acid-supplemented medium (Fig. 4c). Similarly, plasma membrane pore formation and pyroptosis were identical in the *Atg7*^*f/f*^ and *Atg7*^*M*∆^ macrophages as measured by PI uptake, LDH release, and GSDMD and caspase-1 processing (Supplementary Fig. 12c–e).

The decreased IL-1β release by neutrophils from *Atg7*^*M*∆^ mice phenocopied the responses observed in neutrophils from *Gsdmd*^*−/−*^ mice, *i.e*., inhibition of IL-1β secretion following canonical inflammasome activation, intracellular retention of p17 IL-1β, and no difference in total pro-IL-1β accumulation or generation of active caspase-1 (Fig. 4d, e). ASC oligomerization was also identical in neutrophils from C57BL/6 and *Gsdmd*^*−/−*^ mice (Supplementary Fig. 12b).

**Fig. 5:**
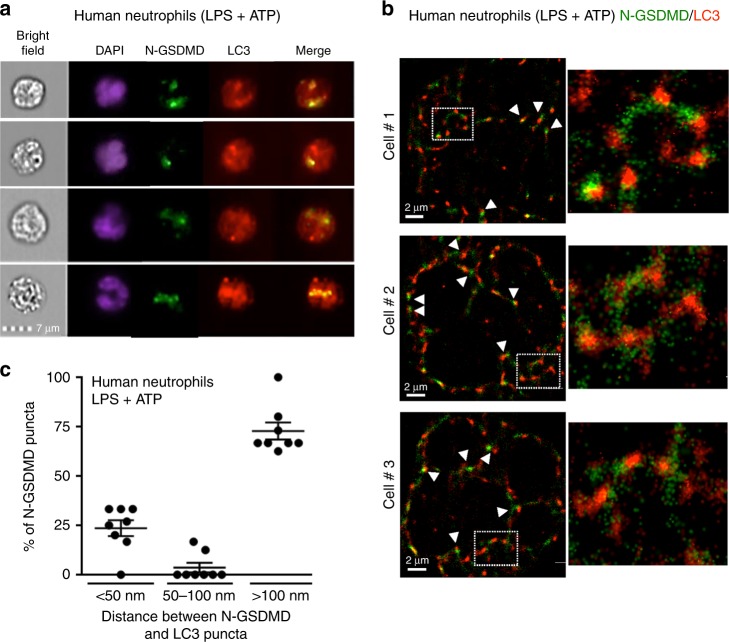
Localization of neutrophil N-GSDMD to LC3^+^ autophagosomes. **a, b** LPS + ATP activated human peripheral blood neutrophils immunostained with antibodies to N-GSDMD (green) and LC3 (red). Representative images were acquired by Imagestream showing overlapping N-GSDMD and LC3 staining (**a**) and by super resolution STORM images (**b**) showing close proximity (indicated by arrowheads) of N-GSDMD (green) and LC3 (red). Enlarged images of boxed areas are shown in the panels on the right. Scale bar = 2 µm. **c** Quantification of N-GSDMD puncta proximity to LC3 puncta. Each data point represents one neutrophil (median of 8 foci measured per cell), and percent of puncta where N-GSDMD is <50 nm, 50–100 nm, or >100 nm from LC3. Imagestream data are representative of at least 3 independent experiments from human blood donors (*n* = 3). Source data for panel C is provided in the separate Source Data file.

To determine if N-GSDMD associates with neutrophil autophagosomes, LPS/ATP-stimulated human neutrophils were co-stained with antibodies to N-GSDMD and LC3 for single cell Imagestream analysis and super-resolution microscopy. We detected overlap of N-GSDMD with LC3 puncta by Imagestream (Fig. 5a), and STORM imaging revealed close proximity (<50 nm) between approximately 25% N-GSDMD puncta with LC3 (Fig. 5b, c). Highlighted regions of three representative cells identified LC3^+^/N-GSDMD^+^ vesicles are consistent with N-GSDMD integration into the membrane of autophagosomes (Fig. 5b, right panels).

**Fig. 6:**
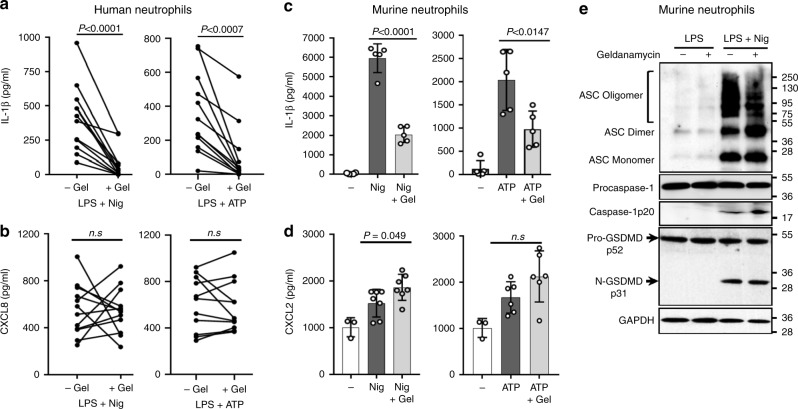
Geldanamycin inhibition of NLRP3 inflammasome-induced IL-1β release. **a**–**d** Human peripheral blood neutrophils (*n* = 12 donors) or bone marrow neutrophils from C57BL/6 mice were LPS primed (3 h), followed by 45 min stimulation with nigericin or ATP in the presence or absence of the HSP-90 inhibitor geldanamycin (Gel, 10 μM). Secretion of IL-1β (**a**, **c**), CXCL8 (**b**), or CXCL2 (**d**) was quantified by ELISA. **e** Western blots of ASC oligomers, caspase-1 and GSDMD from LPS/nigericin-stimulated murine bone marrow neutrophils ±10 μM geldanamycin. Western blot data are representative of 3 independent experiments. Molecular weight markers in kDa are indicated on the right. For human neutrophils, each data point represents 1 donor and *p*-values were based on paired *t* tests from 12 donors (*n* = 12). For murine neutrophils, means±SD from at least 5 independent experiments are shown as individual data points (*n* = 5). *p*-values for murine neutrophils were derived by unpaired *t* test using Tukey post analysis; *p* < 0.05: significant, n.s.: not significant. Source data for all western blots in panel **e** are provided as uncropped blots in Supplementary Information, Supplementary Fig. 1^9^. Source data for panels **a**, **c**, **b**, **d** are provided in the separate Source Data file.

To examine further if there is a role for autophagy in IL-1β secretion from neutrophils, we inhibited cargo loading into the autophagy machinery using the Hsp90 inhibitor geldanamycin. Schekman and colleagues demonstrated that geldanamycin blocked Hsp90 chaperone-assisted transport of mature IL-1β into autophagosome vesicle intermediates in an engineered HEK293 cell model of autophagy-assisted IL-1β secretion^28^. Peripheral blood human neutrophils from 12 donors and murine neutrophils were incubated with geldanamycin after LPS priming, but immediately before NLRP3 activation by nigericin or ATP. IL-1β secretion was significantly inhibited in geldanamycin-treated neutrophils from each human donor (Fig. 6a), and inhibition was not a consequence of impaired cell viability as there was no effect on secretion of CXCL8/IL-8 via the conventional ER-Golgi pathway (Fig. 6b). Geldanamycin-treated murine neutrophils also released significantly less IL-1β than controls, whereas there was no suppression of CXCL2 secretion (Fig. 6c, d). Moreover, geldanamycin treatment did not inhibit ASC oligomerization, caspase-1 processing or GSDMD cleavage in NLRP3-activated murine neutrophils (Fig. 6e), indicating that N-GSDMD acts downstream of inflammasome assembly and caspase-1 activation to suppress IL-1β export.

We also induced autophagy by amino acid starvation, and found that starvation further increased IL-1β secretion by NLRP3-activated murine neutrophils in the absence of pyroptotic LDH release, although the effect of amino acid starvation was more variable in human neutrophils, with only 5 out of 9 donors showing elevated IL-1β release (Supplementary Fig. 13a–c).

Overall, these findings are consistent with a role for GSDMD in an autophagy machinery-dependent IL-1β secretion mechanism by neutrophils that is independent of plasma membrane N-GSDMD pore formation and pyroptosis.

## Discussion

Since GSDMD was identified as the mediator of caspase-1 dependent IL-1β release and pyroptosis^10,29^, most studies have focused on the roles of plasma membrane (PM) GSDMD pores and consequent pyroptotic cell lysis as the primary mechanism for IL-1β release from macrophages. The major findings in our study are that cleaved N-GSDMD generated during NLRP3 inflammasome signaling in neutrophils predominantly associates with the membranes of abundant intracellular organelles rather than the PM. This absence of PM N-GSDMD pores additionally reflects reduced rates of caspase-1 activation and N-GSDMD accumulation in neutrophils relative to macrophages during canonical inflammasome activation.

**Fig. 7:**
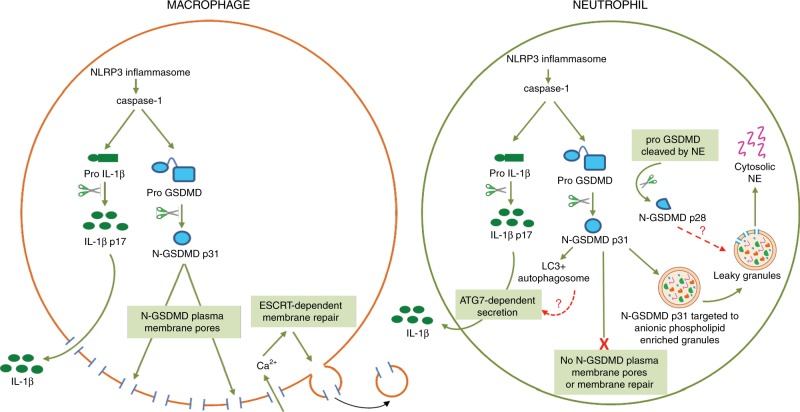
Proposed model for GSDMD signaling in inflammasome activated neutrophils and macrophages. Comparison of NLRP3 inflammasome signaling and downstream GSDMD trafficking in neutrophils and macrophages (see text for detailed discussion).

Results of the current study showing differences in the role of GSDMD in neutrophils compared with macrophages are presented in Fig. 7. Canonical NLRP3 activation of macrophages results in N-GSDMD mediated release of IL-1β through increased plasma membrane permeability, membrane repair and pyroptosis. In contrast, alternative trafficking of a smaller pool of N-GSDMD in neutrophils has three major consequences for inflammasome signaling in these granulocytic leukocytes. First, it greatly reduces accumulation of functional N-GSDMD pores in the neutrophil plasma membrane, and thus provides an underlying mechanism for the absence of neutrophil pyroptosis. Second, it results in N-GSDMD pore formation in azurophilic granules with consequent release of granule contents, such as neutrophil elastase, into the cytosol; in turn, the cytosolic elastase drives a secondary phase of GSDMD proteolytic processing. Third, it is coordinated with engagement of autophagy signaling such that mature IL-1β is secreted from neutrophils via a non-canonical, non-lytic pathway dependent on key elements of the canonical autophagy machinery. Importantly, our functional analyses indicated that the absence of GSDMD-dependent pyroptosis in neutrophils is not due to Ca^2+^-influx-dependent plasma membrane repair, which is in contrast to macrophages^18^.

Our observation that p31 N-GSDMD associates with intracellular organelle membranes rather than the plasma membrane in inflammasome-activated neutrophils adds to the growing literature describing GSDMD-dependent subcellular perturbations, including mitochondrial permeabilization and reduced motility that can precede or is uncoupled from pyroptotic cell death^30–32^. Thus, cell type-specific thresholds for inflammatory activation, non-lytic cell death or pyroptosis during proteolytic activation of GSDMD likely depend on its trafficking and the relative dynamics and extent of N-GSDMD pore formation in intracellular membrane compartments and organelles. In neutrophils, pro-GSDMD cleavage by caspases and/or serine proteases will depend on the strength or nature of the stimulus. For example. bacterial infection and consequent superoxide generation may induce accumulation of sufficient N-GSDMD to overcome the ‘granule sink’ and thereby form pores in the plasma membrane. This explanation is consistent with recently described roles for GSDMD in other regulated neutrophil functions, including neutrophil extracellular trap formation (NETosis) at early and late stages of this process^33,34^, and constitutive (or spontaneous) death of senescent neutrophils^23^. GSDMD is cleaved by neutrophil elastase released from azurophilic granules during phorbol ester-induced superoxide production to drive a feed-forward cascade of enhanced granule and plasma membrane permeabilization required for NETosis^33^. In an alternative model of NETosis, activation of caspase-11 by cytosolic LPS triggered rapid production of cleaved N-GSDMD in amounts sufficient to permeabilize both the nuclear and plasma membrane compartments for efficient externalization of DNA traps^34^.

Constitutive death of aging neutrophils also involves gradual loss of azurophilic granule membrane integrity and cytosolic accumulation of granule serine proteases^35^. Recent studies demonstrated that neutrophil elastase efficiently cleaves human and murine GSDMD at 15 or 25 residues (respectively) upstream of the canonical caspase-1 cleavage site (D275-Hu/ D276-Mu) to yield a smaller (28–29 kDa) N-GSDMD fragment, which is also pore-competent^23^. We extended these observations by showing that initial accumulation of caspase-1 cleaved p31-N-GSDMD in neutrophils facilitates secondary GSDMD processing by elastase to generate p28-N-GSDMD. However, in our model of NLRP3 inflammasome signaling, neutrophils did not progress to lytic death even after 8 h of sustained activation. As is standard for analyses of NLRP3 inflammasome signaling, we used neutrophils primed with LPS to induce expression of proIL-1β and upregulate NLRP3. LPS treatment also markedly delays spontaneous neutrophil death^36,37^. In a broader physiological context, the absence of GSDMD-mediated pyroptosis during inflammasome signaling also preserves neutrophil viability required for direct bacterial killing, while still allowing neutrophil IL-1β release into tissue compartments to sustain the inflammatory environment until bacteria are cleared. This concept is supported by the delayed role of elastase-mediated GSDMD cleavage in controlling senescent neutrophil death^23^.

Because IL-1β lacks a signal sequence for conventional secretion, non-canonical export is required to deliver the cytosolic pool of mature IL-1β to extracellular compartments. Several mechanisms have been suggested (reviewed in refs. ^38–40^), including: (1) accumulation of IL-1β within membrane-bound subcellular compartments (exosomes, microvesicles, secretory lysosomes) for export to the extracellular space in the absence of lysis; (2) pre-lytic efflux of IL-1β via active plasma membrane GSDMD pores; and (3) IL-1β release as a passive consequence of GSDMD-dependent pyroptosis. It is unclear whether the membrane compartmentalization pathways operate independently of GSDMD, or whether they are regulated (directly or indirectly) by GSDMD.

Schroder and colleagues reported that IL-1β release from macrophages involves the localization of cleaved IL-1β in PIP2- rich microdomains of the inner leaflet of the plasma membrane mediated by positively charged amino acids of IL-1β, followed by its release through either a rapid N-GSDMD pore-mediated path or a slower GSDMD-independent mechanism likely involving microvesicle shedding^17^. However, autophagy-relevant proteins have also been implicated in non-canonical secretion of IL-1β from macrophages and neutrophils^41–43^. Our current finding that ATG7-deficient murine neutrophils exhibit impaired IL-1β secretion further supports a critical role for the autophagy machinery in this process. These results are consistent with those of Zhang et al. who used an HEK293 reporter system to show that IL-1β is transported by autophagosomes, and requires association of IL-1β with Hsp90 to traffic into autophagosomes prior to fusion with the plasma membrane^28^. Although we clearly detect N-GSDMD in LC3^+^ vesicles, the mechanism by which GSDMD regulates autophagy or ATG7-dependent IL-1β secretion has yet to be determined. It is possible that N-GSDMD facilitates loading of IL-1β into autophagosomes or that N-GSDMD contributes to the fusion of autophagosomes with the plasma membrane rather than lysosomes by interacting with specific cargo loading proteins. These hypotheses are supported by the similar phenotypes shown for suppressed IL-1β secretion in *Atg7*^*M*∆^ and *Gsdmd*^−/−^ neutrophils, and are consistent with a role for GSDMD in an autophagy-dependent IL-1β secretion by neutrophils. However, we cannot eliminate the possibility that the localization of N-GSDMD on LC3^+^ vesicles is independent of IL-1β secretion.

It is also relevant that the functions of other Gasdermin family proteins have been linked to autophagy signaling. Expression of either N-GDSMA3 or a gain-of-function mutant pro-GSDMA3 in HEK293 cells induced increased autophagy and accumulation of LC3-II^44^. Expression of autosomal recessive mutations in Pejvakin (PJVK, also known as DFNB59 or GSDMF) is associated with death of inner hair cells and underlies hearing impairments in mice or humans with such PJVK mutations^45,46^. Normal PJVK recruits LC3-II to the membrane of damaged peroxisomes to drive their clearance by selective autophagy (pexophagy); this protective function is ablated by the PJVK mutations associated with hearing loss^47^.

In macrophages, canonical macro-autophagy acts to restrain inflammasome signaling and IL-1β production by directing ubiquitinated inflammasome complexes and pro-IL-1β to lysosomes for degradation^25,40,48–50^. Given the predominant role for canonical plasma membrane GSDMD pores in mediating IL-1β release from macrophages, discrimination of the specific contribution from autophagy-dependent secretion to total export of IL-1β from monocyte/macrophages likely varies with the particular mode and duration of inflammasome activation stimuli, as well as metabolic conditions that suppress or induce autophagy.

Neutrophils have abundant primary, secondary and tertiary granules, and other membrane bound organelles such as autophagosomes compared with mononuclear leukocytes. As insertion of N-GSDMD into the plasma membrane requires lipid bilayers enriched in phosphoinositides or other anionic phospholipids, we suggest that the predominant insertion of N-GSDMD into membranes of intracellular organelles will both restrain pore accumulation in the plasma membrane, and shape the innate immune functions of those organelles. Together, these parameters describe the fundamental differences in GSDMD trafficking between neutrophils and macrophages that underlie neutrophil-specific functions during inflammasome signaling in infection and responses to sterile tissue damage.

## Materials and methods

### Reagents

All reagents and antibodies are listed in Supplementary Information Table 1.

### Source of mice

C57BL/6 and neutrophil elastase (Ne^*−/−*^) mice on a C57BL/6 background were purchased from The Jackson Laboratory (Bar Harbor, ME), and bred at Case Western Reserve University and UC Irvine. *Gsdmd*^*−/−*^ mice were generated by Dr. Russell Vance (University of California, Berkeley) on a C57BL/6 background as described^51^, and were bred at UC Irvine. Myeloid specific deletion of *Atg7* (*Atg7*^MΔ^) was obtained by breeding *Atg7*^f/f^ mice (parent strain) with LysM-Cre transgenic mice (from Jackson Laboratory) as described^27^, and bones were sent to us by Dr. Tony Eissa (Baylor College of Medicine, Texas). All animals were housed in pathogen free conditions in microisolator cages and were treated according to institutional guidelines following approval by the Case Western Reserve University and the University of California IACUC.

### Primary murine neutrophils

Total bone marrow cells were isolated from tibias and femurs, and neutrophils were purified using the EasySep™ Mouse Neutrophil Enrichment Kit (Stem Cell Technologies), which works by negative selection using magnetic beads. This process routinely yielded >94% pure neutrophils (by flow cytometry of Ly6G+/CD11b+ cells) as we described^15^.

### Human neutrophils

Whole blood was collected from healthy donors between ages 18 and 65 years in accordance with the Declaration of Helsinki guidelines and the Institutional Review Board of the University of California (Irvine, CA). Written, informed consent was obtained from each donor by the Institute for Clinical and Translational Science at UC Irvine, and was de-identified prior to use in this project. Neutrophils were then isolated using Ficoll-Paque Plus (GE Healthcare) by density gradient centrifugation at 300 × *g* for −0min, which yields >90% purity as assessed by flow cytometry using anti-human CD16 and CD66b Abs (eBiosciences).

### Murine and human macrophages

For primary macrophages, total bone marrow cells were isolated, and differentiated into macrophages for 7 days using MCSF in the culture media as described^11^. After differentiation, primary and immortalized murine macrophages were cultured in high glucose DMEM supplemented with 10% FBS, 2mM L-Glutamine in presence of penicillin and streptomycin. Human THP-1 monocytes were cultured in RPMI 1640 supplemented with 10% FBS, 2mM L-Glutamine and penicillin and streptomycin. *Gsdmd*^−/−^ THP-1 cells were generated using CRISPR-Cas9 as described^52^.

### NLRP3 inflammasome priming and activation

Human and murine neutrophils were incubated (primed) with 500 ng/ml LPS for 3 hr at 37 °C in RPMI-1640 media containing 2% FBS. Following priming, cells were stimulated with the NLRP3 inflammasome activators nigericin, ATP or *Streptococcus pneumoniae* pneumolysin. Murine macrophages were primed with LPS (1 µg/ml) for 3 hr at 37 °C in DMEM media containing 2% FBS. Chemical inhibitors were added to primed cells for 30 minutes prior to adding inflammasome activators. Cell-free extracellular media were collected for ELISA or for TCA precipitation of secreted proteins as described previously.

### ELISA for cytokine quantification

Half-well cytokine assays were performed using Duoset ELISA assay kits for murine and human IL-1β, murine CXCL2 and human CXCL8/IL-8 according to manufacturer’s protocols (R&D Systems).

### Plasma membrane pore formation by propidium influx

Cells were plated in 24-well plates (0.5 million cells/well for macrophages and 1million cells/well for neutrophils). After LPS priming, culture medium was removed and the cells were washed with PBS before adding basal salt solution (BSS) [130 mM NaCl, 4 mM KCL, 1.5 mM CaCl_2_, 1 mM MgCl_2_, 25 mM HEPES, 5 mM D-Glucose and 0.1% BSA] supplemented with 1 µg/ml PI. Baseline fluorescence (540 nm excitation → 620 nm emission) was recorded for 5 mins in Synergy HT plate reader (BioTek). Cells were then stimulated using nigericin and ATP, and fluorescence was recorded. As a positive control, cells were permeabilized using 1% Triton X-100. Values are presented as a percentage of Triton X-100 maximum fluorescence in permeabilized cells after subtracting basal intrinsic fluorescence. PI fluorescence and phase contrast images were acquired using a Cytation5 imaging reader (BioTek).

For PI uptake analysis by flow cytometry, neutrophils and macrophages were primed with LPS and stimulated with ATP or nigericin as described. At indicated times after stimulation, cells were harvested, washed once in PBS, blocked with mouse CD16/CD32 Fc block for 10 minutes in FACS buffer, and incubated with Ly6G-FITC or F4/80-FITC for 15 minutes at 4°C. Cells were washed once in FACS buffer, resuspended in PBS containing 1.5 μM propidium iodide, and analyzed using a Novocyte flow cytometer (ACEA Biosciences).

### Cytotoxicity assay (LDH release)

After stimulation of neutrophils or macrophages, supernatant was collected and LDH release was quantified using CytoTox 96® Non-Radioactive Cytotoxicity Assay (Promega) according to the manufacturer’s instructions. Percentage cytotoxicity was calculated based on maximum LDH release from unstimulated cells lysed with 1% Triton X-100.

### Preparation of macrophage lysates

Whole cell lysates of murine macrophages were prepared by lysis in RIPA buffer (150 mM NaCl, 50 mM Tris-HCl, 1% Triton X-100, 0.5% Na-deoxycholate) supplemented with conventional protease inhibitor cocktail.

### Preparation of neutrophil lysates

Neutrophils contain high levels of serine proteases (neutrophil elastase (NE), cathepsin G (CG), and proteinase 3 (PR3)) stored within the azurophilic granules. These proteases require neutral pH for their catalytic activity which is: (a) basally suppressed by the acidic pH maintained within intact granules; and (b) relatively insensitive to the standard protease inhibitors in lysis buffers used to generate whole cell extracts for western blot or immunoprecipitation. Thus, detergent solubilization of azurophilic granules in neutral pH buffers results in protein cleavage by the catalytic activity of the stored serine proteases, which occurs rapidly post-lysis before neutrophil lysates are denatured by extraction into SDS and heating.

To inhibit post-lysis processing of neutrophil proteins, diisopropylfluorophosphate (DFP), which is a permeable and covalent inhibitor of the neutral serine proteases, was added to the lysates. DFP in RIPA buffer was critical to prevent significant post-lysis processing of GSDMD in neutrophil extracts as demonstrated and discussed in Supplemental Fig. 9. Thus, for all experiments involving western blot analyses of inflammasome and GSDMD signaling, stimulated neutrophils were pelleted by brief centrifugation, removal of test medium supernatant, and immediate extraction of the cell pellet into in RIPA buffer (150 mM NaCl, 50 mM Tris-HCl, 1% Triton X-100, 0.5% Na-deoxycholate) supplemented with 5 mM DFP in addition to the conventional protease inhibitor cocktail.

### Western blot

Proteins in neutrophil and macrophage lysates were resolved in 15% SDS-PAGE, transferred to nitrocellulose membranes, and incubated with primary antibodies to IL-1β, Caspase-1 p20, anti-mouse GSDMD, Tom-20, Pan-cadherin, MPO, ELA-2, LC3, or ATPB1. Total protein was assessed using antibodies to β-actin or GAPDH. Reactivity was determined using HRP-conjugated secondary antibodies (Santa Cruz) and developed using Supersignal West Femto Maximum Sensitivity Substrate (Pierce).

### Subcellular fractionation of neutrophils and macrophages

Macrophages were homogenized in ice cold fractionation buffer (250 mM sucrose, 20 mM HEPES pH 7.4, 10 mM KCl, 1.5 mM MgCl_2_, 1 mM EDTA, 1 mM EGTA, protease inhibitor cocktail and 5 mM DFP). Neutrophils were subjected to N_2_ cavitation (to ensure integrity of intracellular granules) at 400 psi for 10 mins in the same fractionation buffer. The homogenate/cavitate was then centrifuged at 720 *g*, 10k *g* and 100k *g* to obtain distinct subcellular fractions.

### Isolation of cytosolic fractions

Stimulated neutrophils were homogenized in ice cold fractionation buffer (250 mM sucrose, 20 mM HEPES pH 7.4, 10 mM KCl, 1.5 mM MgCl_2_, 1 mM EDTA, 1 mM EGTA, protease inhibitor cocktail and 5 mM DFP). The homogenates were centrifuged at 720 *g* and 10,000 *g* to remove nuclei and larger organelles, respectively. The supernatant was then centrifuged at 16,000 *g* for 30 min to isolate the cytosolic fraction, which was then TCA precipitated and subject to SDS-PAGE and western blot.

### Amnis Imagestream flow cytometry and co-localization analyses

Following activation of human neutrophils, cells were fixed with 4% paraformaldehyde for 10 min, and incubated with FITC conjugated wheat germ agglutinin (L4895, Sigma-Aldrich) to identify the plasma membrane. Cells were then permeabilized with 0.1% Triton X-100, and blocked with 10% normal donkey serum, 0.5% BSA, 2 mM EDTA and 0.02% TX-100 in PBS for 1 hr at 4 °C. Neutrophils were incubated overnight with rabbit anti-human N-GSDMD antibody (EPR20829-408), anti-human/mouse MPO or anti-human LC3 antibody at 1:25 dilution. Cells were then washed and incubated with Alexa Fluor 488 anti-rabbit, Alexa Fluor 647 anti-mouse or 5Alexa Fluor 594 anti-goat secondary antibodies (Life Technologies). Samples were mounted on slides using VECTASHIELD mounting media containing DAPI (VectorLabs), and were examined by Imagestream (Amnis).

### Super resolution microscopy (STORM imaging)

Cells were labeled with primary antibodies to human LC3 or MPO and secondary Alexa 647-anti-rat, or with rabbit anti-human N-GSDMD and secondary Atto488-anti-rabbit, and resuspended in freshly prepared STORM buffer as described in detail^53^. Samples were imaged on a Nikon Ti super resolution microscope using a 100×/1.49 NA Apo TIRF objective either with IXON3 ultra DU897 electron—multiplying CCD camera using multi-color sequential mode setting in the Nikon Elements software. STORM images (cropped regions of interest containing single cells) were reconstructed and analyzed using the General Analysis (GA) module in the Nikon Elements software suite. Briefly, after applying appropriate threshold and filtering out noise, the minimum distance of the GSDMD puncta to the closest LC3/MPO vesicle was measured using the MinDistance tool under GA. The results were binned into three categories: <50 nm (close proximity), 50–100 nm and >100 nm. For each cell, the distances from each GSDMD puncta to MPO or LC3 puncta were used to build a histogram. Eight neutrophils were analyzed for N-GSDMD/LC3 proximity (median of 9 foci per cell) and 23 neutrophils for N-GSDMD/MPO proximity (median of 12 foci per cell).

### Elastase assay

Quantification of neutrophil elastase activity was measured using a fluorescence based assay from BioVision, which provides purified enzyme and a fluorescent substrate to generate a standard curve.

### Statistics

Student *t*-test or ANOVA with Sidak’s multiple comparisons analysis (Prism, Graphpad Software) were used as indicated in the figure legends. Paired Student *t*-test was used for individual donor neutrophils±inhibitors. A *p*-value equal or less than 0.05 was considered significant.

## Supplementary information

Supplementary Information

## Data Availability

The authors declare that data supporting the findings of the current study are available within the article files and Supplementary Information or available from the corresponding authors upon request. The flow cytometry gating strategies and uncropped versions for all the western blot images are included in the Supplementary Information. The source data underlying all the bar graphs, scatter plots and kinetic studies in the article and supplementary information are provided in Excel spreadsheets within a separate Source Data file.

## Source Data

Source Data

## References

[1] Broz, P., Pelegrin, P. & Shao, F. The gasdermins, a protein family executing cell death and inflammation. *Nat. Rev. Immunol.*10.1038/s41577-019-0228-2 (2019). 10.1038/s41577-019-0228-2 31690840

[2] Orning P, Lien E, Fitzgerald KA (2019). Gasdermins and their role in immunity and inflammation. J. Exp. Med..

[3] Shi J, Gao W, Shao F (2017). Pyroptosis: gasdermin-mediated programmed necrotic cell death. Trends Biochem. Sci..

[4] Kovacs SB, Miao EA (2017). Gasdermins: effectors of pyroptosis. Trends Cell Biol..

[5] Sborgi L (2016). GSDMD membrane pore formation constitutes the mechanism of pyroptotic cell death. EMBO J..

[6] Liu X (2016). Inflammasome-activated gasdermin D causes pyroptosis by forming membrane pores. Nature.

[7] Ding J (2016). Pore-forming activity and structural autoinhibition of the gasdermin family. Nature.

[8] Jorgensen I, Zhang Y, Krantz BA, Miao EA (2016). Pyroptosis triggers pore-induced intracellular traps (PITs) that capture bacteria and lead to their clearance by efferocytosis. J. Exp. Med..

[9] Dinarello CA (2011). Interleukin-1 in the pathogenesis and treatment of inflammatory diseases. Blood.

[10] Shi J (2015). Cleavage of GSDMD by inflammatory caspases determines pyroptotic cell death. Nature.

[11] Russo HM (2016). Active caspase-1 induces plasma membrane pores that precede pyroptotic lysis and are blocked by lanthanides. J. Immunol..

[12] Evavold CL (2018). The pore-forming protein gasdermin D regulates interleukin-1 secretion from living macrophages. Immunity.

[13] Chen KW (2014). The neutrophil NLRC4 inflammasome selectively promotes IL-1beta maturation without pyroptosis during acute Salmonella challenge. Cell Rep..

[14] Karmakar M, A KM, Dubyak GR, Pearlman E (2016). Neutrophil P2X7 receptors mediate NLRP3 inflammasome-dependent IL-1β secretion in response to ATP. Nat. Commun..

[15] Karmakar M (2015). Neutrophil IL-1beta processing induced by pneumolysin is mediated by the NLRP3/ASC inflammasome and caspase-1 activation and is dependent on K+ efflux. J. Immunol..

[16] Heilig R (2018). The gasdermin-D pore acts as a conduit for IL-1beta secretion in mice. Eur. J. Immunol..

[17] Monteleone M (2018). Interleukin-1beta maturation triggers its relocation to the plasma membrane for gasdermin-D-dependent and -independent secretion. Cell Rep..

[18] Ruhl S (2018). ESCRT-dependent membrane repair negatively regulates pyroptosis downstream of GSDMD activation. Science.

[19] Ruan J, Xia S, Liu X, Lieberman J, Wu H (2018). Cryo-EM structure of the gasdermin A3 membrane pore. Nature.

[20] Man SM, Karki R, Kanneganti TD (2017). Molecular mechanisms and functions of pyroptosis, inflammatory caspases and inflammasomes in infectious diseases. Immunol. Rev..

[21] Lamkanfi M (2008). Targeted peptidecentric proteomics reveals caspase-7 as a substrate of the caspase-1 inflammasomes. Mol. Cell Proteom..

[22] Taabazuing CY, Okondo MC, Bachovchin DA (2017). Pyroptosis and apoptosis pathways engage in bidirectional crosstalk in monocytes and macrophages. Cell Chem. Biol..

[23] Kambara H (2018). Gasdermin D exerts anti-inflammatory effects by promoting neutrophil death. Cell Rep..

[24] Burgener SS (2019). Cathepsin G inhibition by serpinb1 and serpinb6 prevents programmed necrosis in neutrophils and monocytes and reduces GSDMD-driven inflammation. Cell Rep..

[25] Kimura T (2017). Dedicated SNAREs and specialized TRIM cargo receptors mediate secretory autophagy. EMBO J..

[26] Dupont N (2011). Autophagy-based unconventional secretory pathway for extracellular delivery of IL-1beta. EMBO J..

[27] Bhattacharya A (2015). Autophagy is required for neutrophil-mediated inflammation. Cell Rep..

[28] Zhang M, Kenny SJ, Ge L, Xu K, Schekman R (2015). Translocation of interleukin-1beta into a vesicle intermediate in autophagy-mediated secretion. eLife.

[29] Kayagaki N (2015). Caspase-11 cleaves gasdermin D for non-canonical inflammasome signalling. Nature.

[30] de Vasconcelos NM, Van Opdenbosch N, Van Gorp H, Parthoens E, Lamkanfi M (2019). Single-cell analysis of pyroptosis dynamics reveals conserved GSDMD-mediated subcellular events that precede plasma membrane rupture. Cell Death Differ..

[31] Rogers C (2019). Gasdermin pores permeabilize mitochondria to augment caspase-3 activation during apoptosis and inflammasome activation. Nat. Commun..

[32] DiPeso L, Ji DX, Vance RE, Price JV (2017). Cell death and cell lysis are separable events during pyroptosis. Cell Death Discov..

[33] Sollberger G (2018). Gasdermin D plays a vital role in the generation of neutrophil extracellular traps. Sci. Immunol..

[34] Chen KW (2018). Noncanonical inflammasome signaling elicits gasdermin D-dependent neutrophil extracellular traps. Sci. Immunol..

[35] Teng Y, Luo HR, Kambara H (2017). Heterogeneity of neutrophil spontaneous death. Am. J. Hematol..

[36] Luo HR, Loison F (2008). Constitutive neutrophil apoptosis: mechanisms and regulation. Am. J. Hematol..

[37] Adrover JM, Nicolas-Avila JA, Hidalgo A (2016). Aging: a temporal dimension for neutrophils. Trends Immunol..

[38] Dubyak GR (2012). P2X7 receptor regulation of non-classical secretion from immune effector cells. Cell Microbiol.

[39] Sitia R, Rubartelli A (2018). The unconventional secretion of IL-1beta: Handling a dangerous weapon to optimize inflammatory responses. Semin Cell Dev. Biol..

[40] Claude-Taupin A, Bissa B, Jia J, Gu Y, Deretic V (2018). Role of autophagy in IL-1beta export and release from cells. Semin Cell Dev. Biol..

[41] Galluzzi L, Green DR (2019). Autophagy-independent functions of the autophagy machinery. Cell.

[42] Semino C, Carta S, Gattorno M, Sitia R, Rubartelli A (2018). Progressive waves of IL-1beta release by primary human monocytes via sequential activation of vesicular and gasdermin D-mediated secretory pathways. Cell Death Dis..

[43] Iula L (2018). Autophagy mediates interleukin-1beta secretion in human neutrophils. Front. Immunol..

[44] Shi P (2015). Loss of conserved Gsdma3 self-regulation causes autophagy and cell death. Biochem J..

[45] Delmaghani S (2015). Hypervulnerability to sound exposure through impaired adaptive proliferation of peroxisomes. Cell.

[46] Collin RW (2007). Involvement of DFNB59 mutations in autosomal recessive nonsyndromic hearing impairment. Hum. Mutat..

[47] Defourny J (2019). Pejvakin-mediated pexophagy protects auditory hair cells against noise-induced damage. Proc. Natl. Acad. Sci. USA.

[48] Harris J (2011). Autophagy controls IL-1beta secretion by targeting pro-IL-1beta for degradation. J. Biol. Chem..

[49] Shi CS (2012). Activation of autophagy by inflammatory signals limits IL-1beta production by targeting ubiquitinated inflammasomes for destruction. Nat. Immunol..

[50] Katsnelson MA, Lozada-Soto KM, Russo HM, Miller BA, Dubyak GR (2016). NLRP3 inflammasome signaling is activated by low-level lysosome disruption but inhibited by extensive lysosome disruption: roles for K+ efflux and Ca2+ influx. Am. J. Physiol. Cell Physiol..

[51] Nichols RD, von Moltke J, Vance RE (2017). NAIP/NLRC4 inflammasome activation in MRP8(+) cells is sufficient to cause systemic inflammatory disease. Nat. Commun..

[52] Rathkey JK (2017). Live-cell visualization of gasdermin D-driven pyroptotic cell death. J. Biol. Chem..

[53] Johnson JL (2016). Munc13-4 Is a Rab11-binding protein that regulates Rab11-positive vesicle trafficking and docking at the plasma membrane. J. Biol. Chem..

