# Supplementary material for: N-GSDMD trafficking to neutrophil organelles facilitates IL-1β release independently of plasma membrane pores and pyroptosis
Source: Nat Commun. 2020 May 5;11:2212. doi: 10.1038/s41467-020-16043-9 (PMC7200749; doi:10.1038/s41467-020-16043-9)
Supplement: Supplementary file 1 — Supplementary Information [file 41467_2020_16043_MOESM1_ESM.pdf]

N-GSDMD trafficking to neutrophil organelles facilitates IL-1β release independently of plasma membrane pores and pyroptosis.

Karmakar et al.

Supplemental Data

Table 1: Reagents and antibodies

List of Supplementary Figures 1-23

Supplementary Figure 1. *GSDMD dependent IL-1β secretion in neutrophils and macrophages*

Supplementary Figure 2. *Propidium uptake and LDH release responses to ATP or nigericin in macrophages and neutrophils from C57Bl/6 versus Gsdmd$^{-/-}$ mice.*

Supplementary Figure 3. *IL-1β secretion and LDH release from NLRP3 activated human neutrophils.*

Supplementary Figure 4. *IL-1β secretion by murine macrophages and neutrophils stimulated with nigericin in Ca^{2+}- supplemented versus Ca^{2+}- free media.*

Supplementary Figure 5. *Propidium uptake responses in murine macrophages and neutrophils stimulated with pneumolysin pore-forming exotoxin.*

Supplementary Figure 6A. *No difference in pro-GSDMD in neutrophils versus macrophages (A7 antibody).*
Supplementary Figure 6B. *p28 N-GSDMD in nigericin stimulated macrophages is a product of Caspase-3/7 cleavage*.

Supplementary Figure 7. *Negative control for N-GSDMD staining.*

Supplementary Figure 8. *Subcellular fractions of p31 N-GSDMD in organelle (**Org**), plasma membrane (**PM**) and cytosolic fractions (**Cyto)** of LPS/Nig stimulated bone marrow neutrophils and bone marrow derived macrophages.* ***B.*** *Plasma membrane and cytosolic fractions of bone marrow neutrophils stimulated with LPS only.* ***C, D.*** *Caspase- p20, IL-1β and GSDMD in cell supernatants of LPS/ATP or LPS/Nig stimulated macrophages and neutrophils*

Supplementary Figure 9*. Neutrophil elastase (NE) cleavage of pro-GSDMD*; Discussion on following page

Supplementary Figure 10*. GSDMD processing in murine macrophages in the presence of DFP or bafilomycin A.*

Supplementary Figure 11. *Neutrophil viability during prolonged inflammasome activation*

Supplementary Figure 12. A, B*. ASC oligomerization and caspase-1 cleavage in LPS/Nigericin – stimulated WT, Gsdmd$^{-/-}$ and Atg7$^{-/-}$ neutrophils.*
C, D*. PI uptake, LDH release and western blot analysis from NLRP3-activated Atg7$^{f/f}$ (WT) and Atg7$^{M\Delta}$ bone marrow derived macrophages*
E. *GSDMD and caspase-1 processing by macrophages from WT and Atg7$^{M\Delta}$ (KO) mice*

Supplementary Figure 13. *Enhanced IL-1β secretion by neutrophils in amino acid starvation media*

Supplementary Figure 14. *Gating strategy for flow cytometry.*

Supplementary Figures 15-23*. Uncropped western blot images*

Supplementary Table 1: Reagents and antibodies

Reagents	Source	Working Concentration
Ultra-pure LPS (*E. coli* K12)	Invivogen	1μg/ml for macrophages<br>500ng/ml for neutrophils
Nigericin	Tocris	10μM
ATP	Sigma Aldrich	3mM
Recombinant pneumolysin (ply)	Gift from Aras Kadioglu	500ng/ml
zVAD	APExBio	50μM
MCC 950	Invivogen	10μM
Bafilomycin A	APExBio	0.5μM
Propidium Iodide	Life Technologies	1.5μM
Geldanamycin	APExBio	10μM
Diisopropylflurophosphate (DFP)	Sigma Aldrich	5mM
Rabbit anti-mouse GSDMD mAb (clone EPR 19828)	Abcam	1:1000 (western blot)
Mouse monoclonal anti-mouse GSDMD mAb (clone A7)	Santa Cruz	1:250 (western blot)
Mouse monoclonal anti-human GSDMD mAb (clone H-6))	Santa Cruz	1:250 (western blot)
Goat anti-mouse/human MPO polyclonal Ab (AF3667)	R&D Systems	0.5μg/ml (western blot)
Rat anti-mouse ELA2 mAB (Catalog# MAB4517)	R&D Systems	0.5μg/ml (western blot)
Goat anti-mouse/human IL-1β polyclonal Ab (Catalog# AF401)	R&D Systems	0.5μg/ml (western blot)
Rat anti-mouse caspase-1 (Catalog# 645102)	Biolegend	2μg/ml (western blot)
Rabbit anti-mouse Tom-20 (Catalog#11415)	Santa Cruz	1:1000 (western blot)
Rabbit anti-mouse/human pan cadherin polyclonal Ab (Catalog# 4068)	Cell Signaling Technologies	1:1000 (western blot)
Rabbit anti-mouse/human LC3 polyclonal Ab (Catalog# 8918)	Sigma Aldrich	1:1000 (western blot)
Mouse anti human ATPB1 (clone 3D5)	Abcam	0.5μg/ml (western blot)
Anti-GAPDH-HRP conjugate (Catalog# MA5-15738-HRP)	Fisher Scientific	1:2000 (western blot)
Anti-β-actin-HRP conjugate (Catalog# A3854)	Sigma Aldrich	1:50,000 (western blot)
HRP-conjugated anti-goat or anti rabbit	Santa Cruz	1:4500 (western blot)
HRP-conjugated anti-mouse or anti rat	Cell Signaling Technologies	1:2000 (western blot)
Rabbit anti-human N-GSDMD monoclonal antibody	Generated by Feng Shao (Now available at Abcam EPR 20829-408)	1:100 (microscopy)<br>1:1000 (western blot)
Mouse anti-human LC3 mAb (clone G9)	Santa Cruz	1:20 (microscopy)
Alexa Fluor 488 anti-rabbit	Life Technologies	1:1000 (microscopy)
Alexa Flour 647 anti-rabbit or anti goat	Life Technologies	1:1000 (microscopy)
FITC anti-mouse Ly-6G Antibody (Catalog # 127605)	Biolegend	1:100 (Flow cytometry)
FITC anti-mouse F4/80 Antibody (Catalog #123107)	Biolegend	1:100 (Flow cytometry)
Fixable Viability Dye eFluor™ 780 (Catalog #65-0865-14)	eBioscience	1:100 (Flow cytometry)

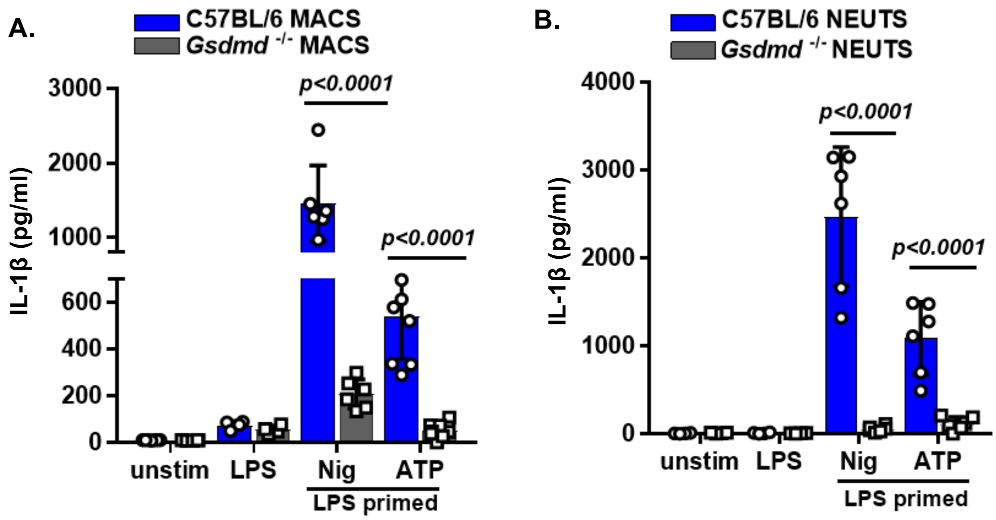

Supplementary Figure 1. ***GSDMD dependent IL-1β secretion in neutrophils and macrophages.***
A- B. Bone marrow derived macrophages (**A**) and bone marrow neutrophils (**B**) from C57BL/6 and *Gsdmd*$^{-/-}$ mice were LPS-primed (3h) and stimulated for 45mins with 3mM ATP or 10μM nigericin. IL-1β secretion was quantified by ELISA. Data shown are means +/- SD of 6 independent experiments (n=6 data points) with experimental replicates for each condition. Statistical analysis were performed by two-way ANOVA using Sidak's multiple comparisons test (GraphPad prism software). Source data for Panels A, B are provided in the separate Source Data file.

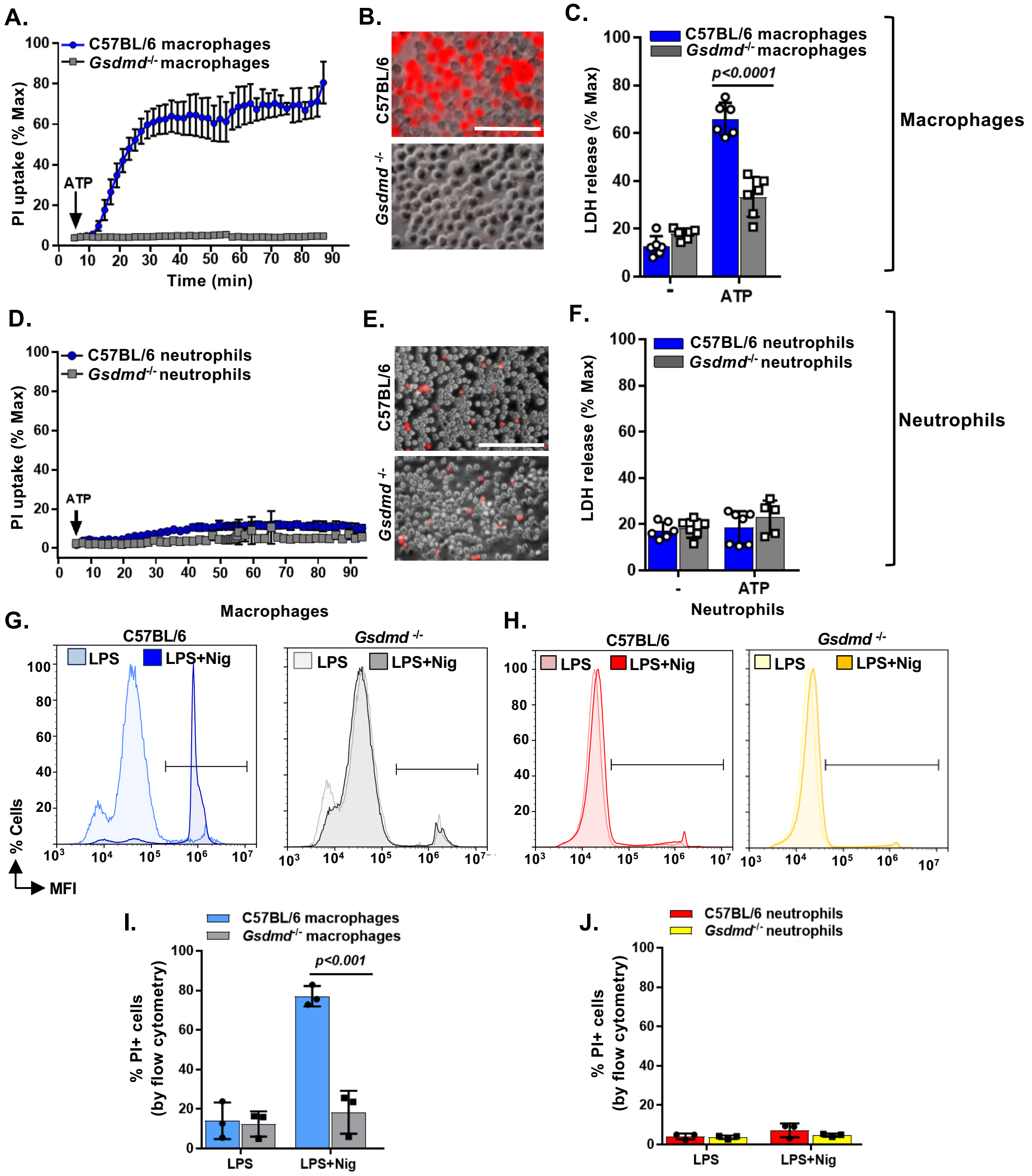

Supplementary Figure 2. ***Propidium uptake and LDH release responses to ATP or nigericin in macrophages and neutrophils from C57Bl/6 versus Gsdmd$^{-/-}$ mice***.

Bone marrow derived macrophages (**A- C**) and bone marrow neutrophils (**D- F**) from C57BL/6 and *Gsdmd*$^{-/-}$ mice were LPS-primed and stimulated for 45 min with 3mM ATP. Propidium iodide (1.5µM) was added to the cells and PI fluorescence was measured over time. Phase contrast images (**B, E**) were captured after 45 min. Scale bar is 100µm. In A and B, macrophages were imaged in the presence of 5mM glycine to inhibit cell lysis. **C, F**. LDH release from ATP-stimulated macrophages and neutrophils after 90mins incubation with ATP. **G- J***.* Representative flow cytometry plots (**G, H**) and % of PI-positive C57BL/6 and *Gsdmd*$^{-/-}$ macrophages and neutrophils after 45min stimulation with nigericin (nig). Data in **A**, **D** are representative of four independent experiments (n=4) with triplicate wells for each condition. LDH data (**C**, **F**) are mean +/- SD of means of 5 independent experiments (n=5 data points). Data in **G, H** are representative of 3 independent experiments (n=3). **I, J**. Data are mean +/- SD of 3 independent repeat experiments (n=3). *p values* were derived by two-way ANOVA using Sidak's multiple comparisons test (GraphPad Prism software). Details of methods and flow cytometry gating strategy are in Supplemental Figure 14. Source data for Panels A, C, D, F, I, J are provided in the separate Source Data file. FACS gating protocol for panels G, H is provided in Supplementary Information, Supplementary Figure 14..

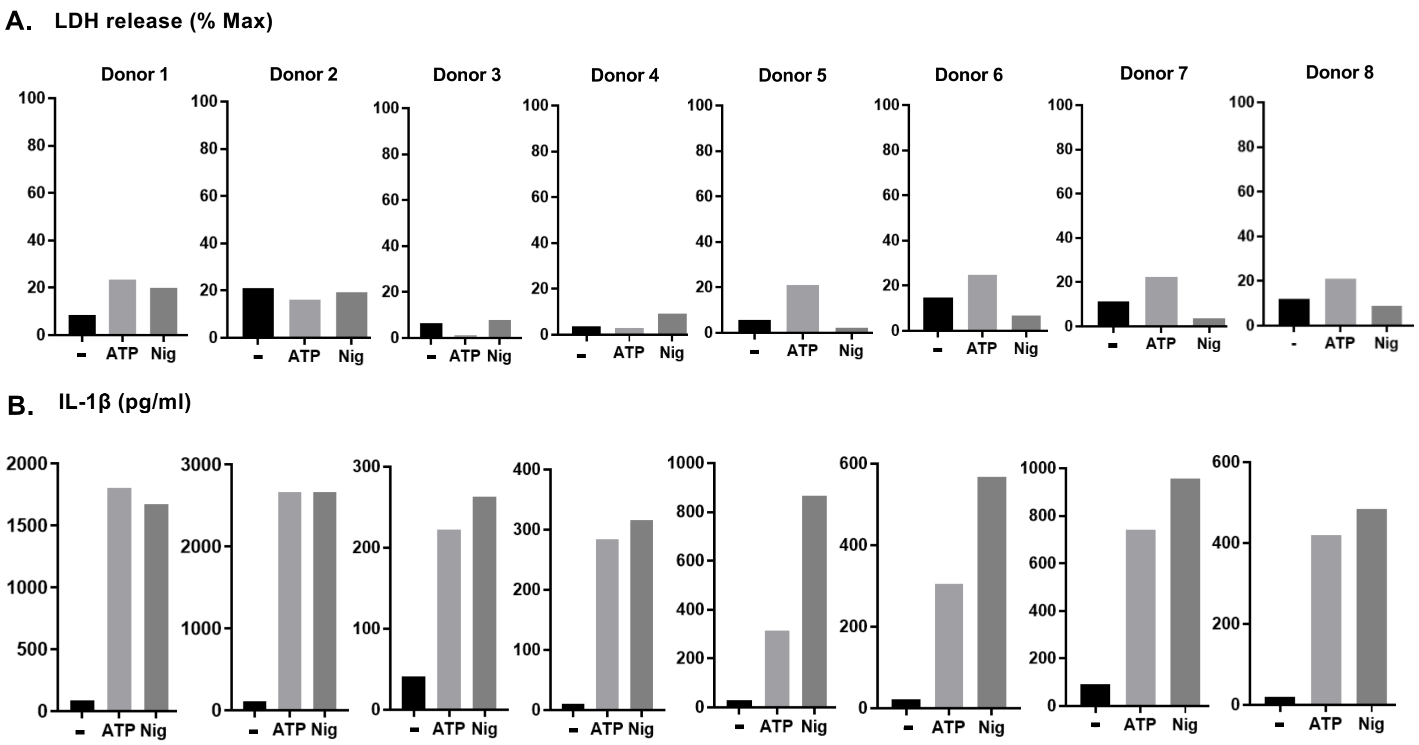

Supplementary Figure 3. ***IL-1β secretion and LDH release from NLRP3 activated human neutrophils*.**
A. Peripheral blood neutrophils from eight different healthy donors were LPS primed for 3h followed by ATP (3mM) and nigericin (10μM) treatment for 45mins. Supernatants were collected for quantification of LDH **(A)** and IL-1β **(B)** release. Data are mean of 4 technical replicates for each donor. Source data for all pnales are provided in the separate Source Data file.

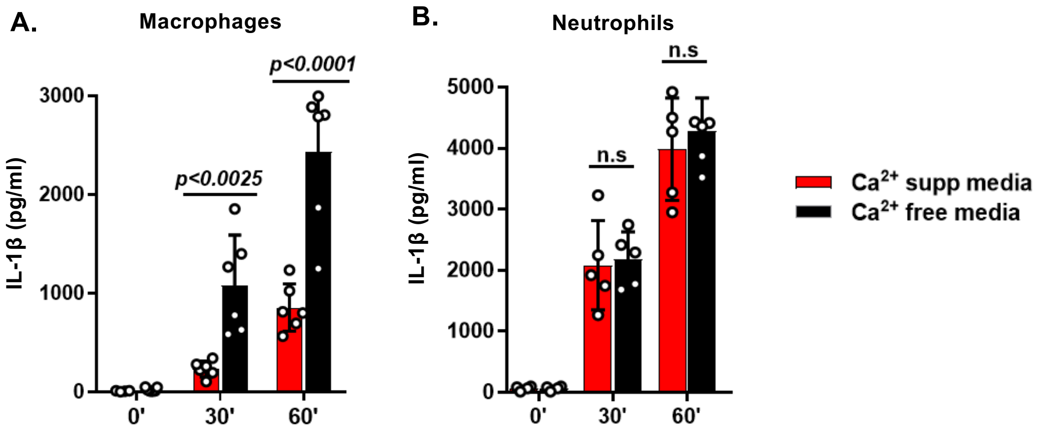

Supplementary Figure 4. ***IL-1β secretion by murine macrophages and neutrophils stimulated with nigericin in Ca^{2+}- supplemented versus Ca^{2+}- free media.***

A, B. Bone marrow derived macrophages and neutrophils were primed with 500ng/ml LPS for 3h followed by stimulation with 10μM nigericin for the indicated time points either in Ca^{2+} supplemented basal salt media (BSS) or in Ca^{2+} free BSS. IL-1β in cell supernatants was quantified by ELISA. Data shown are means +/- SD of 6 independent experiments (n=6 data points) for **A** and 5 independent experiments (n=5) for **B.** Statistical analyses were performed by two-way ANOVA (GraphPad prism software) using Sidak's multiple comparisons test. Source data for Panels A, B are provided in the separate Source Data file.

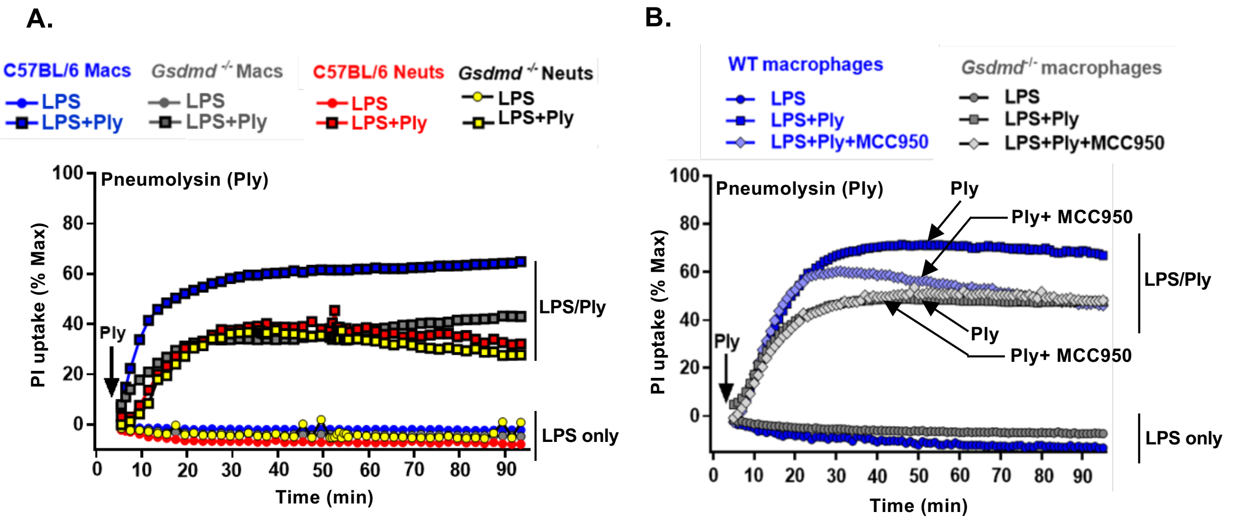

Supplementary Figure 5. ***Propidium uptake responses in murine macrophages and neutrophils stimulated with pneumolysin pore-forming exotoxin.***

A, B. Propidium iodide (PI) uptake following stimulation with sub-lytic concentration of pneumolysin (Ply, 500ng/ml) by macrophages and neutrophils from C57BL/6 or *Gsdmd*$^{-/-}$ mice (**A**) or Ply stimulation of macrophages in presence/absence of 10µM MCC 950 (**B**). Data shown are representative of two independent experiments (n=2). Source data for Panels A, B are provided in the separate Source Data file.

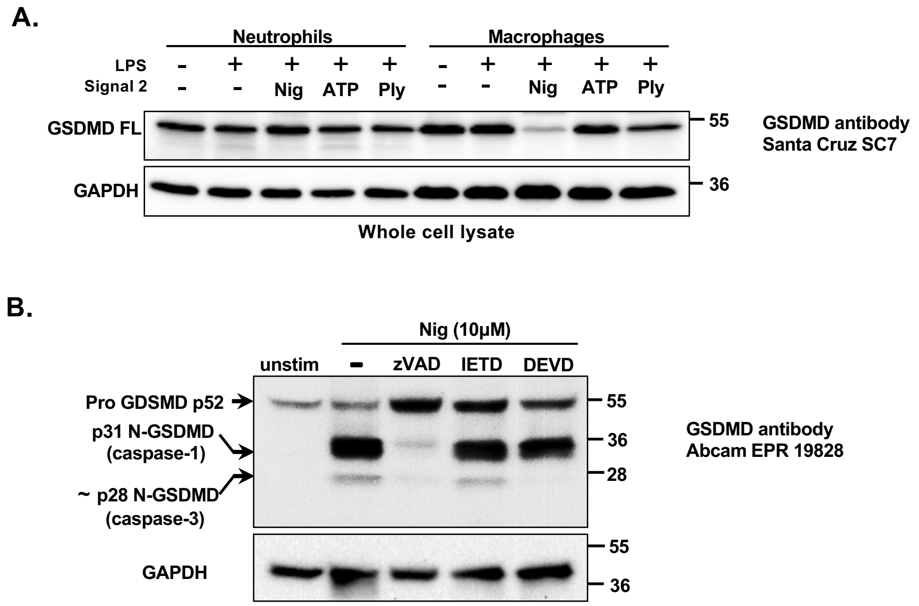

Supplementary Figure 6A. No difference in pro-*GSDMD in neutrophils versus macrophages*. Bone marrow derived macrophages and bone marrow neutrophils were LPS primed and stimulated with 10μM nigericin, 3mM ATP or 500ng/ml pneumolysin for 45mins. 100μg of whole cell lysate were run side-by-side on the same SDS-PAGE and western blot was performed for GSDMD using SC A7 and for GAPDH. Data shown are representative of 3 independent experiments (n=3).

Supplementary Figure 6B. p28 N-*GSDMD in nigericin stimulated macrophages is a product of* Caspase-3/7 cleavage. LPS-primed bone marrow derived macrophages were pretreated for 30mins with pan caspase inhibitor zVAD (50μM), caspase-3/7 inhibitor DEVD (30 μM) and caspase-8 inhibitor IETD (20 μM) and stimulated with nigericin for an additional 45min. Total cell lysates were used to perform western blots for GSDMD (EPR 19828) and GAPDH. Data shown are representative of 2 independent experiments (n=2).

Molecular weight markers in KDa are indicated on the right of the western blot images. Source data for all western blots on Panels A, B are provided as uncropped blots in the Supplementary Information, Supplementary Figure 20.

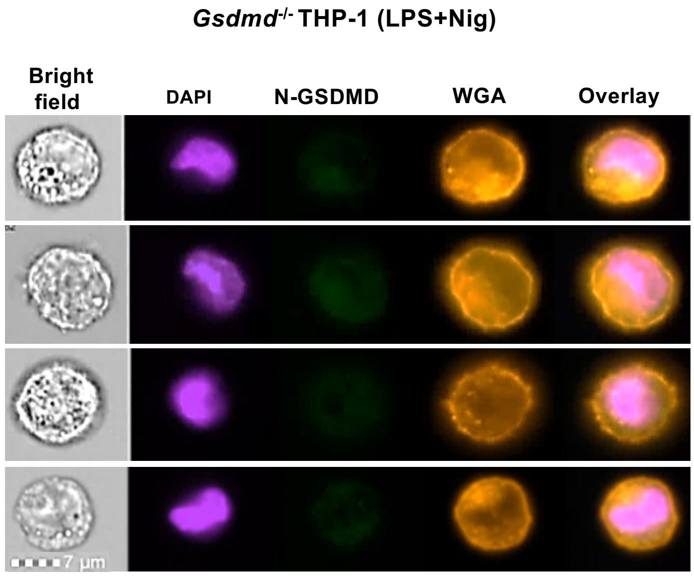

Supplementary Figure 7. ***Negative control for N-GSDMD staining using Abcam*** **EPR 20829-408 antibody*.*** THP1 *Gsdmd*$^{-/-}$ macrophages were LPS primed for 3h and stimulated with 3mM ATP for 45min, and immunostained with N-GSDMD antibody (EPR 20829-408), WGA (wheat germ agglutinin) to stain for plasma membrane and DAPI. Representative Imagestream images showing no N-GSDMD staining in THP1 Gsdmd$^{-/-}$ macrophages (compared with WT THP1 cells shown in Figure 2). Data shown are representative of 4 independent experiments (n=4).

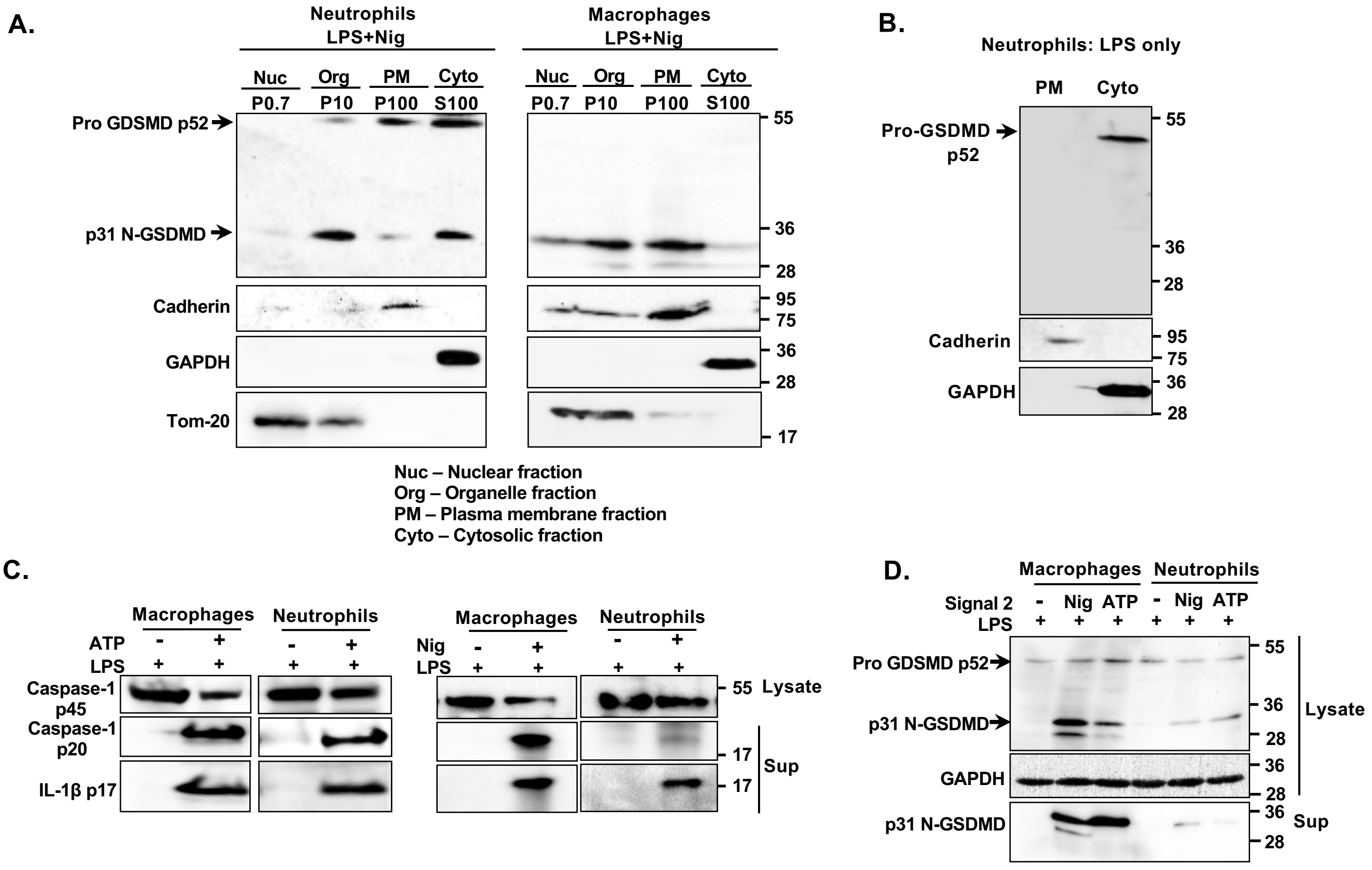

Supplementary Figure 8.

A. Subcellular fractions of p31 N-GSDMD in organelle (**Org**), plasma membrane (**PM**) and cytosolic fractions (**Cyto)** of LPS/Nig stimulated bone marrow neutrophils and bone marrow derived macrophages. **B.** Plasma membrane and cytosolic fractions of bone marrow neutrophils stimulated with LPS only. **C, D.** Caspase- p20, IL-1β and GSDMD in cell supernatants (TCA-precipitation) and lysates following stimulation with LPS/ATP or LPS/Nig. GSDMD westerns used Abcam EPR 19828 antibody. Data are representative of 3 independent experiments (n=3). Molecular weight markers in KDa are indicated on the right of the western blot images. Molecular weight markers in KDa are indicated on the right of the western blot images. Source data for all western blots are provided as uncropped blots in the Supplementary Information, Supplementary Figure 21.

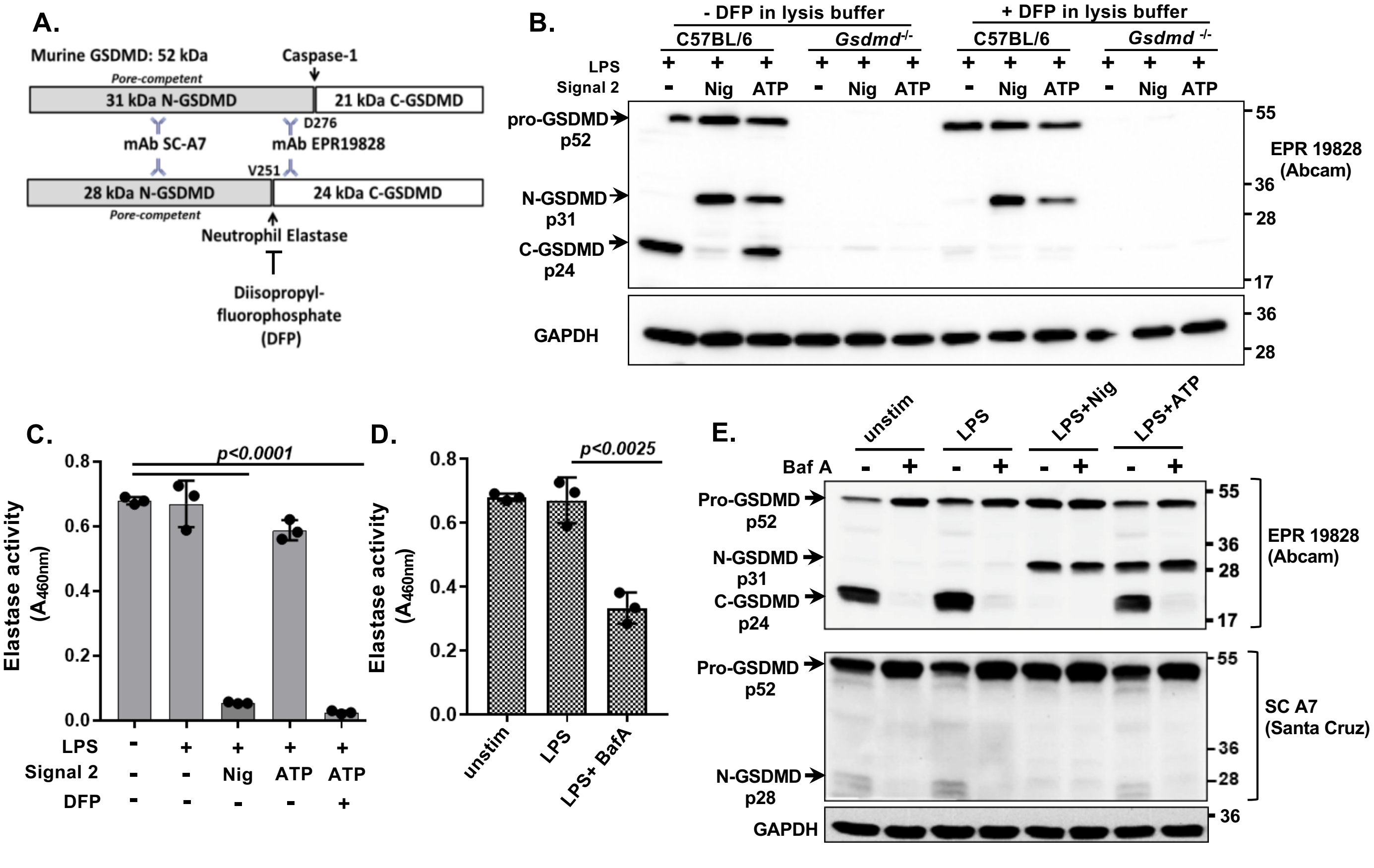

Supplementary Figure 9*. Neutrophil elastase (NE) cleavage of pro-GS*DMD.

A. Cleavage sites for caspase-1 and NE, and GSDMD cleavage products recognized by EPR 19828 (Abcam) and SC-A7 (Santa Cruz) antibodies. **B.** Caspase-1 and NE mediated pro-GSDMD cleavage products in LPS-primed neutrophils 45 min after stimulation with ATP or nigericin (prior to endogenous cleavage by NE). Note that the p24 C-GSDMD is detected in the absence, but not in the presence of the serine protease diisopropylflurophosphate (DFP) in the lysis buffer. p24 was not detected in LPS/Nig stimulated neutrophils (see next page for discussion).

C, D. Enzymatic activity of neutrophil elastase in lysates from neutrophils stimulated with nigericin or ATP in the presence or absence of 5mM DFP **(C),** or 0.5μM bafilomycinA (Baf A) prior to lysis. **E.** Western blot of NLRP3 inflammasome activated neutrophils +/- BafA (0.5μM) using Abcam EPR 19828 or Santa Cruz SC-A7 anti-GSDMD antibodies. Neutrophil lysates for western blot were prepared in the absence of DFP.

Western blots are representative of 3 independent experiments. Molecular weight markers in KDa are indicated on the right of the western blot images. Data in **C** and **D** are mean +/- SD of 3 experiments (n=3 data points). Statistical analyses were performed by unpaired *t* tests using Welch's correction.

Source data for all western blots in Panels B,E are provided as uncropped blots in the Supplementary Information, Supplementary Figure 22; source data for Panels C and D are provided in the separate Source Data File.

See discussion on next page for description of experimental design and discussion of results.

Supplementary Figure 9 Discussion: NE recognizes the V251 upstream of the D276 caspase-1 cleavage site in murine GSDMD to generate a 28kD N-GSDMD product and p24 C-GSDMD fragment. EPR19828 anti-GSDMD mAb from Abcam routinely used in our analyses recognizes the p24 C-GSDMD product generated by NE cleavage in addition to the canonical p31 N-GSDMD produced by caspase-1, but not the p28 N-GSDMD product of NE. In contrast, the SC-A7 anti-GSDMD mAb from Santa Cruz recognizes both the p28 (elastase-derived) and the p31 (caspase-1derived) N-GSDMD cleavage products (**Supplementary Fig 9A**). When LPS-primed neutrophils are detergent-extracted, the 24 kDa C-GSDMD detected by the EPR19828 mAb rapidly accumulates in lysates prepared in DFP-free, but not DFP-supplemented, RIPA buffer (**Supplementary Fig 9B**). When the same comparisons are performed with LPS-primed neutrophils stimulated for 45 min with ATP the p31 N-GSDMD product of caspase-1 cleavage is detected in lysates prepared in the presence or absence of DFP. Notably, an additional p24 C-GSDMD product of NE cleavage is detected only when the cells were lysed in DFP-free RIPA buffer, indicating that this cleavage product is generated post-lysis by NE released from detergent-solubilized azurophilic granules.

We routinely used nigericin and ATP as well-characterized activators of NLRP3 inflammasome signaling in both neutrophils and macrophages. Although nigericin and ATP are equally efficacious inducers of NLRP3 activation and caspase-1 activation in both leukocyte subtypes, we observe mechanistically significant differences in how nigericin and ATP affect the function of azurophilic granules and serine proteases in neutrophils. In contrast to extracts from ATP-stimulated neutrophils, lysates from nigericin-activated neutrophils do not contain the 24kD C-GSDMD product of post-lysis NE cleavage produced in the absence of DFP (**Supplementary Fig 9B**). Thus, nigericin treatment of intact neutrophils results in the irreversible inhibition of the NE stored within azurophilic granules, such that catalytically active NE is not released into detergent extracts to mediate post-lysis cleavage of GSDMD. Nigericin is the most widely used activator of NLRP3 inflammasomes because its activity as a K^+/H^+ exchanging ionophore at the plasma membrane triggers rapid and robust efflux of cytosolic K^+. However, this same K^+/H^+ exchanging activity induces collapse of the H^+ gradients which maintain acidic pH within intracellular organelles such as azurophilic granules of neutrophils and thereby suppress the catalytic activity of the NE stored within the granules. **Supplementary Fig 9C** shows that treatment of neutrophils with nigericin, under conditions used for NLRP3 activation, leads to inactivation of NE catalytic activity. In contrast, NLRP3 activation by ATP does not elicit NE inactivation unless the ATP is co-added with the membrane-permeable DFP.

Thus, an important caveat for experimental analyses of GSDMD function in neutrophils is consideration of how the potent serine proteases within azurophilic granules may be modulated by reagents widely used to manipulate inflammasome and autophagy signaling. Treatment of intact neutrophils with the V-ATPase inhibitor bafilomycin A also results in inactivation of stored NE (**Supplementary Fig 9D**) such that post-lysis cleavage of GSDMD (to the p24-C-terminal or p28 N-terminal products) is prevented even when lysates are prepared in the absence of DFP (**Supplementary Fig 9E**). In contrast, bafilomycin A treatment has no effect on the caspase-1 mediated production of the canonical p31 N-GSDMD. These combined observations demonstrate that maintenance of acidic azurophilic granules is required for activity of the stored serine proteases which can mediate robust cleavage of pro-GSDMD in neutrophils during different types of inflammatory activation.

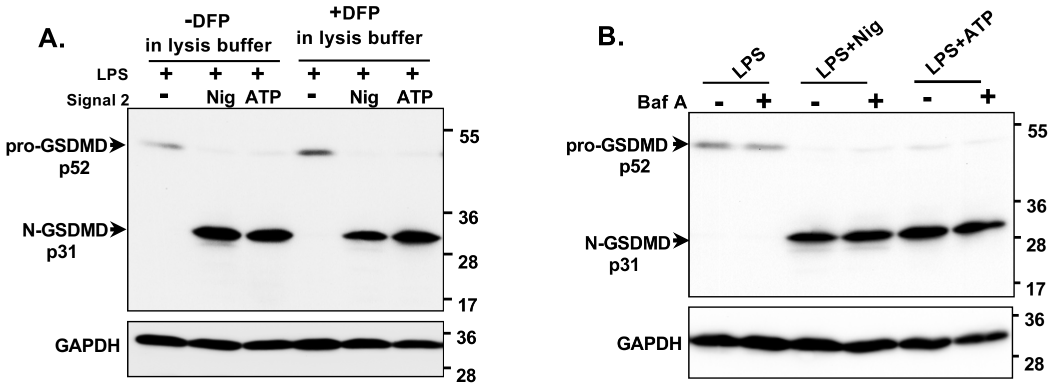

Supplementary Figure 10*. GSDMD processing by murine macrophages is not affected by DFP or bafilomycin A*.

GSDMD western blots (Abcam EPR 19828) of inflammasome-activated murine macrophages +/- 5mM DFP in the lysis buffer (**A)** or +/- BafA (0.5µM) (**B)**. Note that in contrast to neutrophils (Figure 3E, Supplementary Figure 9), there was no p24 C-GSDMD elastase cleavage product in macrophages. Western blots are representative of 2 independent experiments (n=2). Molecular weight markers in KDa are indicated on the right of the western blot images. Source data for all western blots in Panels A, B are provided as uncropped blots in Supplementary Information, Supplementary Figure 22 .

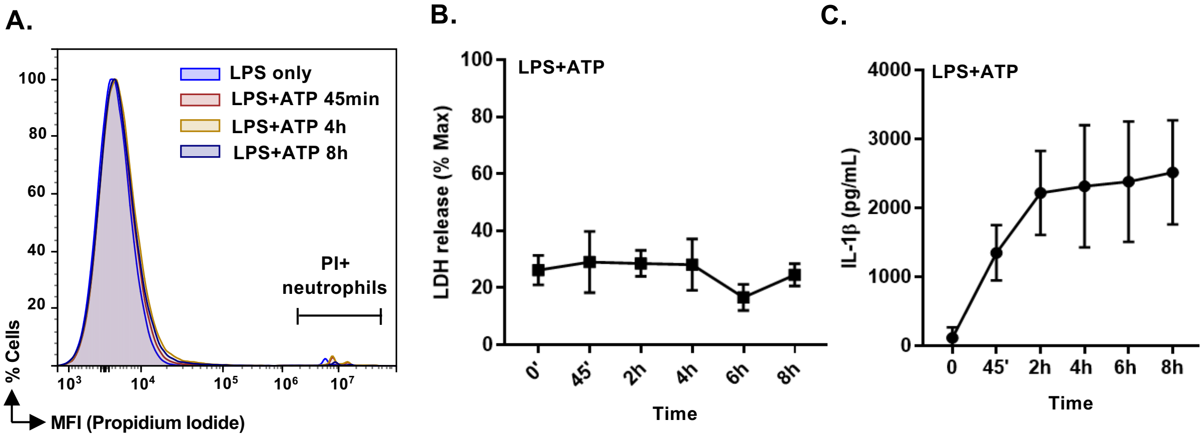

Supplementary Figure 11. ***Neutrophil viability and absence of plasma membrane pore formation during prolonged inflammasome activation with ATP***

Isolated bone marrow neutrophils from C57BL/6 mice were primed with LPS for 3h and stimulated with 3mM ATP for 8h. Membrane permeability was assayed by flow cytometry analysis of propidium iodide uptake (**A**), viability was assessed by LDH release (**B**) and IL-1β secretion was quantified by ELISA (**C**). Flow cytometry data shown are representative of two independent experiments, and LDH and IL-1β ELISA are mean +/- SD of 3 independent experiments with at least 3 replicates per time point. Flow cytometry data is representative of 2 independent experiments (n=2). Source data for Panels B, C are provided in the separate Source Data file. FACS gating protocol for panel A is provided in Supplementary Information, Supplementary Figure 14..

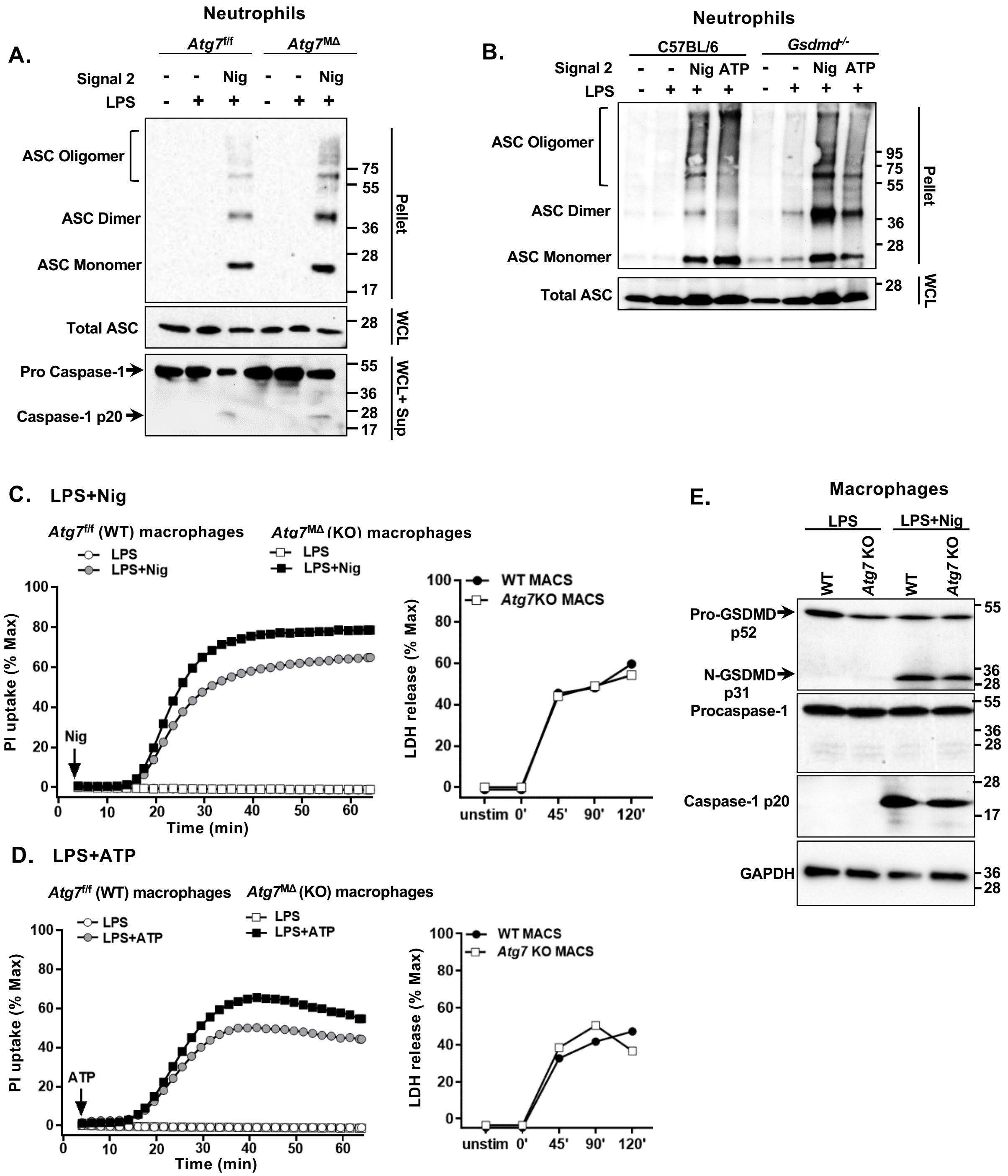

Supplementary Figure 12A,B*. ATG7 and GSDMD are downstream of NLRP3 inflammasome assembly and caspase-1 activation in neutrophils.* ASC oligomerization (from pellet) and caspase-1 cleavage (combined lysate + supernatant) in LPS/nigericin - stimulated neutrophils from A*tg7$^{f/f}$* (WT) and *Atg7$^{M\Delta}$* mice **(A),** and ASC oligomerization from C57BL /6 and *Gsdmd$^{-/-}$* mice **(B)** indicating that the roles of Atg7 and GSDMD are downstream of NLRP3 inflammasome assembly. **WCL**: whole cell lysate, **Sup**: cell supernatant.

Supplementary Figure 12C- E*. ATG7 deficiency in macrophages has no effect on PI uptake, LDH release, or pro-caspase-1 or or pro-GSDMD cleavage.*

PI uptake, LDH release and western blot analysis from NLRP3-activated A*tg7$^{f/f}$* (WT) and *Atg7$^{M\Delta}$* bone marrow derived macrophages. PI uptake over time, and LDH release after 3h LPS priming and stimulation with nigericin (**C**) or ATP (**D**). **E.** GSDMD, caspase-1 and GAPDH western blots from LPS-primed WT and *Atg7$^{M\Delta}$* (KO) macrophages and 45 min stimulation with nigericin.

A, B, E. Western blots are representative of three independent experiments (n=3). Molecular weight markers in KDa are indicated on the right of the western blot images; **C, D**. Data points are mean values from two independent experiments (n=2) with 4 experimental replicates for each condition in each experiment. Source data for all western blots in Panels A, B, and E are provided as uncropped blots in Supplementary Information, Supplementary Figure 23; source data for Panels C and D are provided in the separate Source Data File.

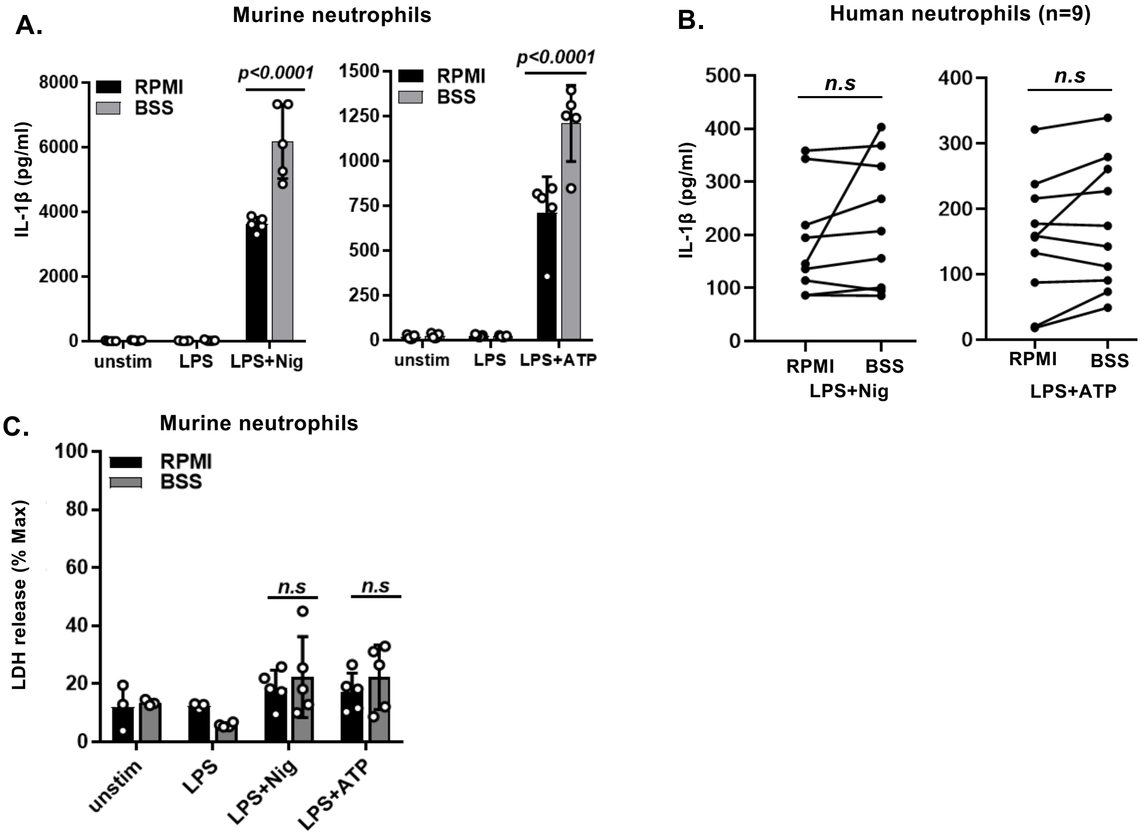

Supplementary Figure 13. ***Enhanced IL-1β secretion by neutrophils in amino acid starvation media***
A, B. IL-1β production by bone marrow neutrophils from C57BL/6 mice (**A**) and from human peripheral blood neutrophils (n=9) **(B)** after LPS priming for 3h followed by 45min stimulation with nigericin or ATP in complete media (**RPMI**) or amino acid starvation media (**BSS**). **C**. LDH release from inflammasome activated murine neutrophils in RPMI and BSS. For **A** and **C**, data presented are +/- SD of mean values from 5 independent experiments (n=5) and statistical analysis were performed by two-way ANOVA using Sidak's multiple comparisons test. For **B**, each data point represents an individual human donor, and statistical analysis was performed using a paired *t* test. Source data for all Panels are provided in the separate Source Data file.

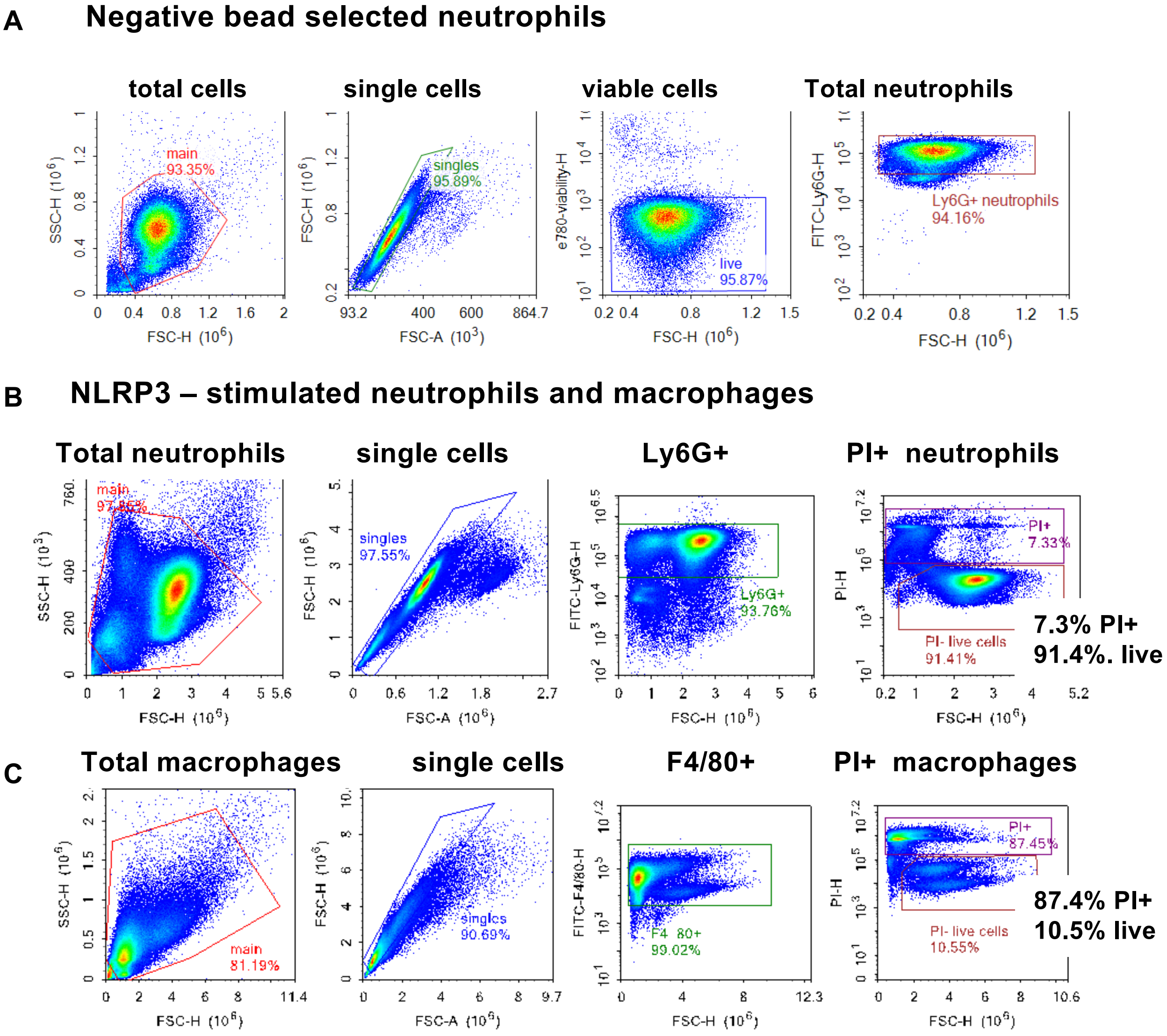

Supplementary Figure 14. ***Flow cytometry gating strategy for PI uptake by neutrophils and macrophages (for Supplementary Fig 2G-J)***

A. Gating strategy for bone marrow neutrophils :
Bone marrow neutrophil gating for purity check following magnetic bead purification: Cells were first gated in FSC-H versus SSC-H to exclude debris. Single cells were then gated based on FSC-A versus FSC-H, and then gated for viable cells based on low fluorescence using the Fixable Viability Dye eFluor™ 780 (eBioscience). Live (eFluor™ 780 -ve), single neutrophils (Ly6G+) were gated based on the viability dye-negative cell population.
Similarly, MCSF – driven bone marrow derived, adherent macrophages were 100% viable prior to NLRP3 stimulation (not shown).
B, C. PI uptake assay: Cells were then primed 3h with LPS and stimulated 45 min with ATP or nigericin. 1μM PI was added to each sample, and examined by flow cytometry using the same gating strategy as in A. In this example, NLRP3 stimulated neutrophils were 7.3% PI positive **(B)**; macrophages were 87.45% PI positive (**C**).

Cells were examined using a NovoCyte Flow cytometer, and NovoExpress software (ACEA Biosciences).

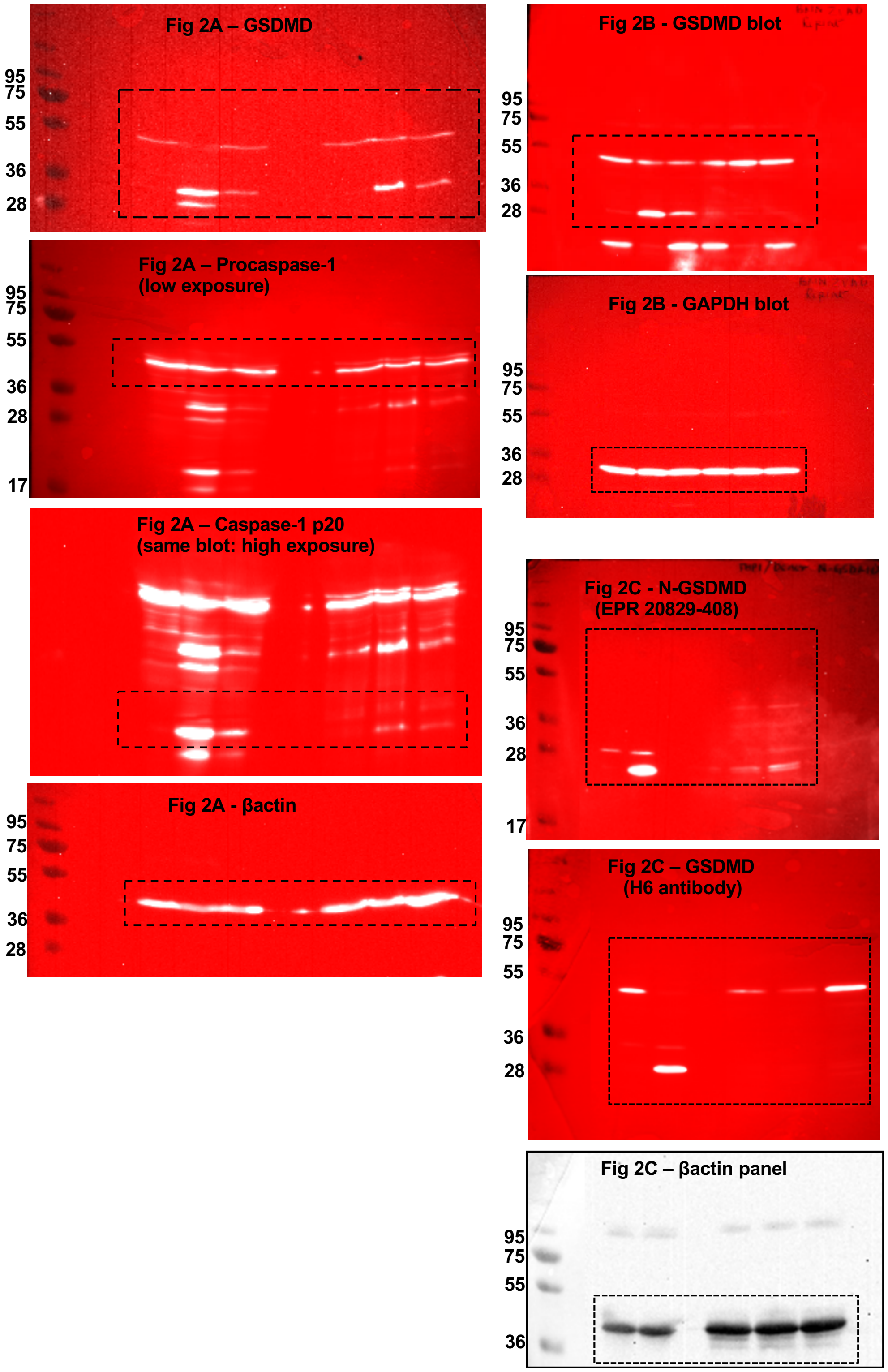

Supplementary Fig 15. ***Un-cropped western blot images for Fig 2***

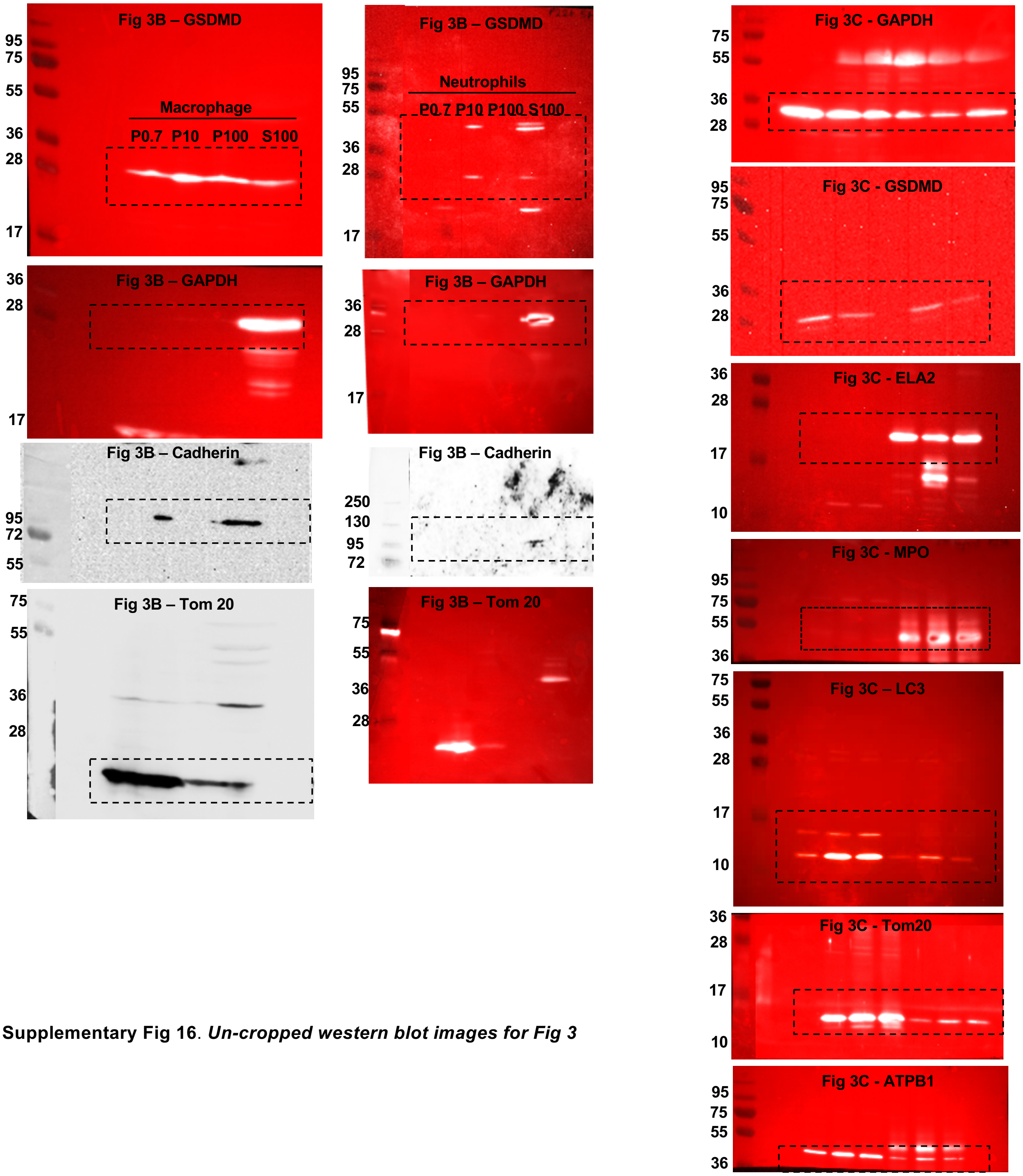

Supplementary Fig 16. ***Un-cropped western blot images for Fig 3***

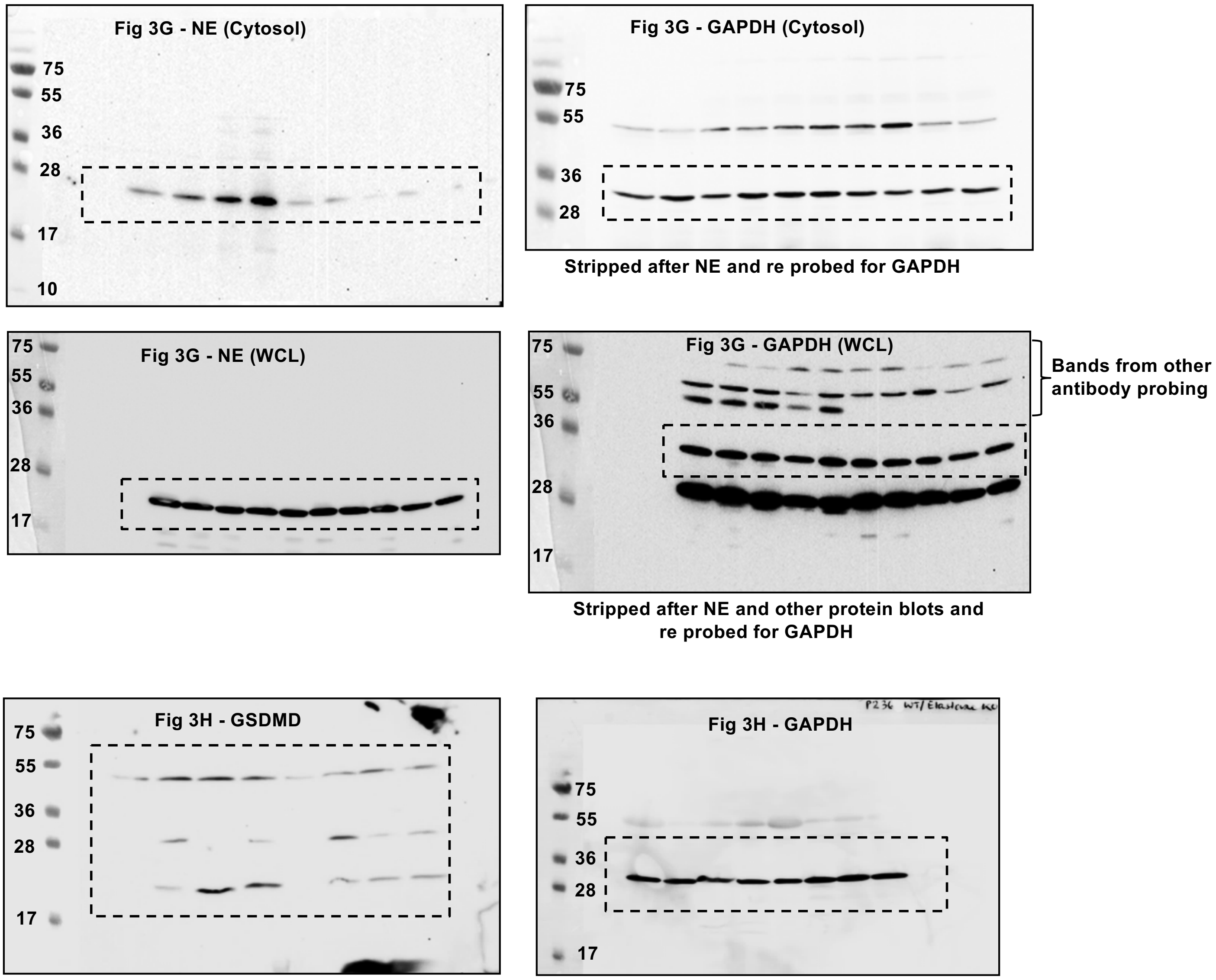

Supplementary Fig 17. ***Un-cropped western blot images for Fig 3 (continued).***

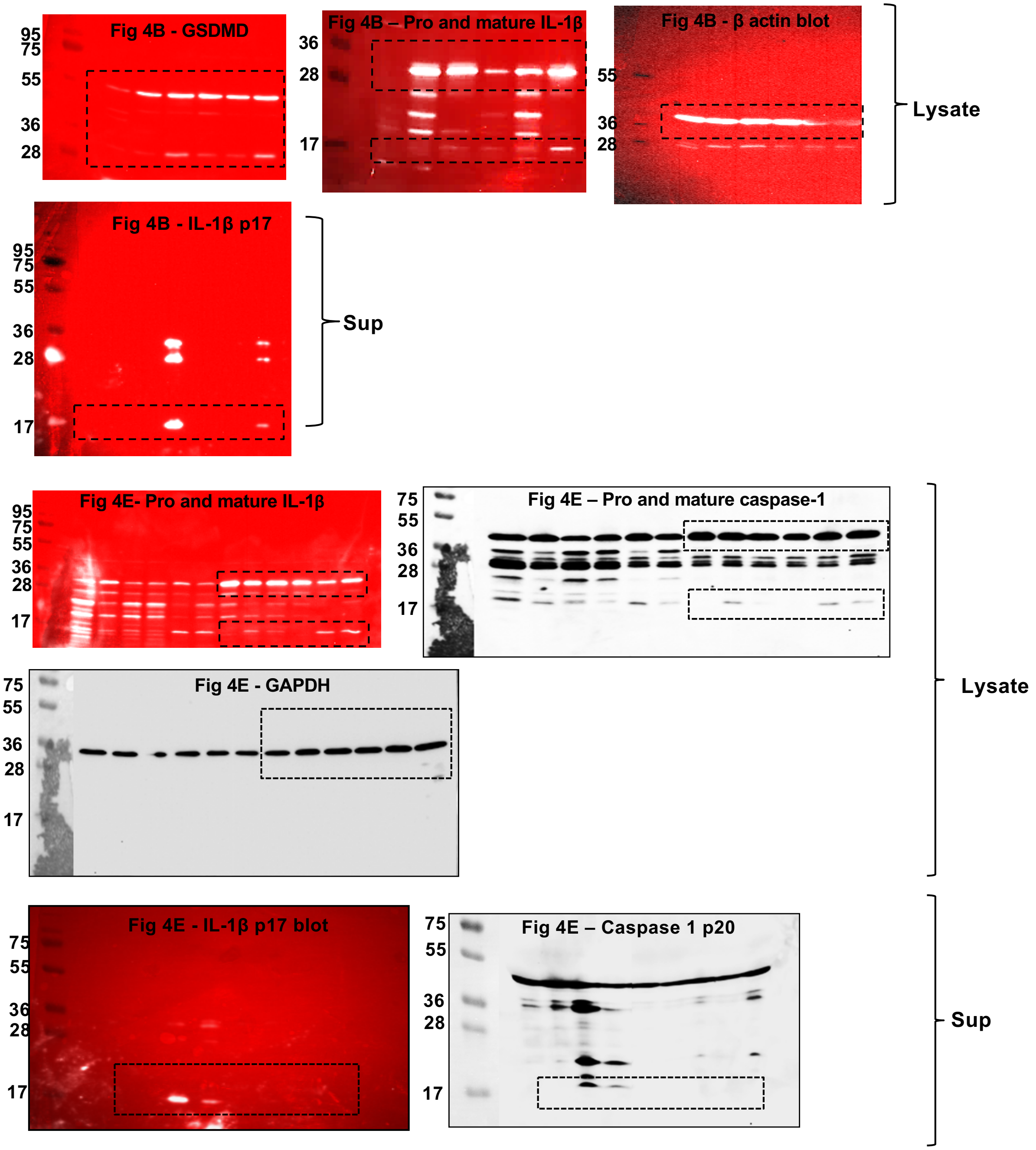

Supplementary Fig 18. *Un-cropped western blot images for Fig 4.*

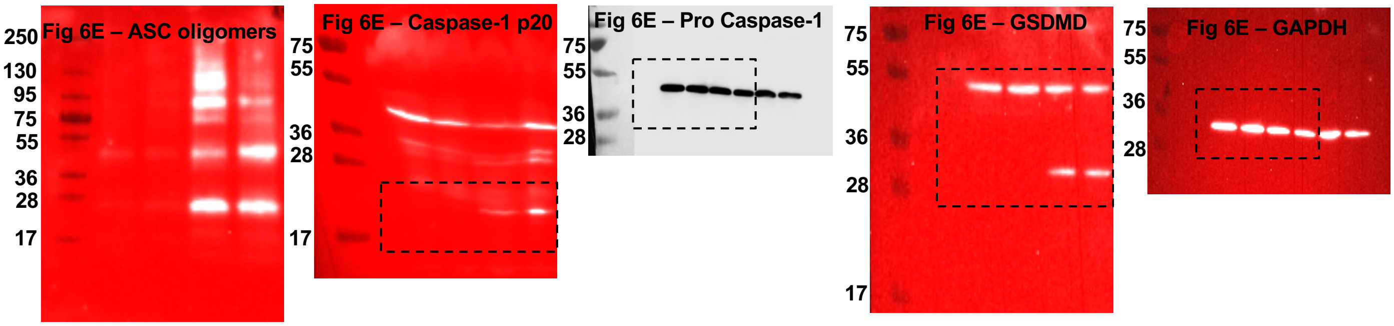

Supplementary Fig 19. ***Un-cropped western blot images for Fig 6.***

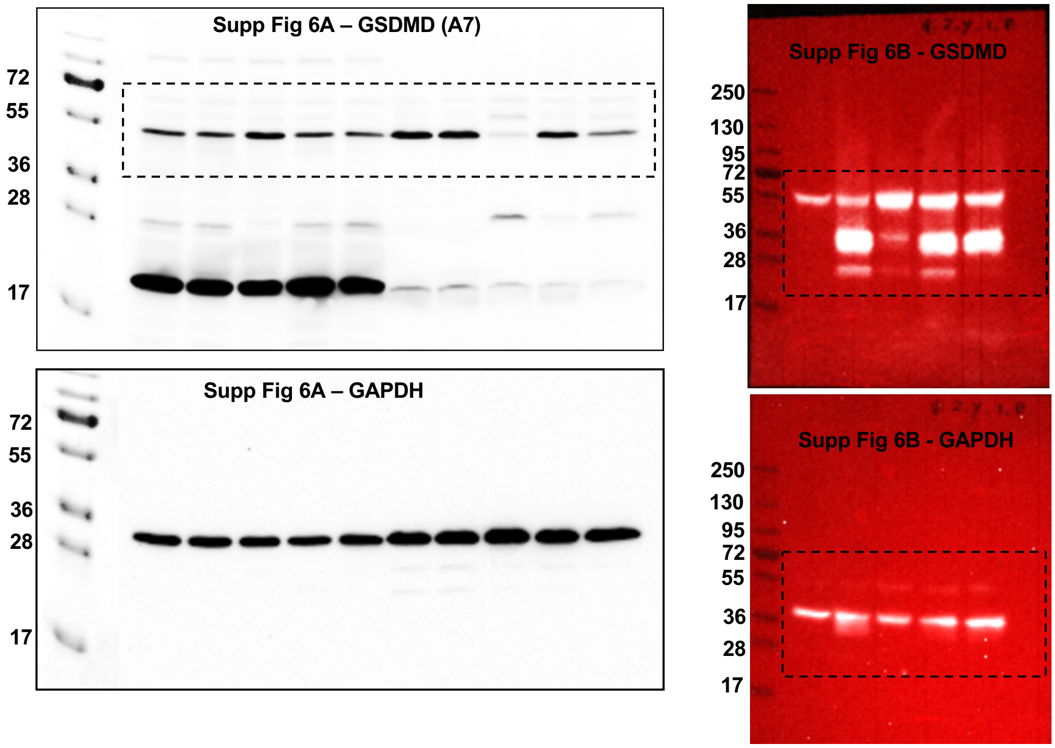

Supplementary Fig 20. ***Un-cropped western blot images for Supplementary Fig 6.***

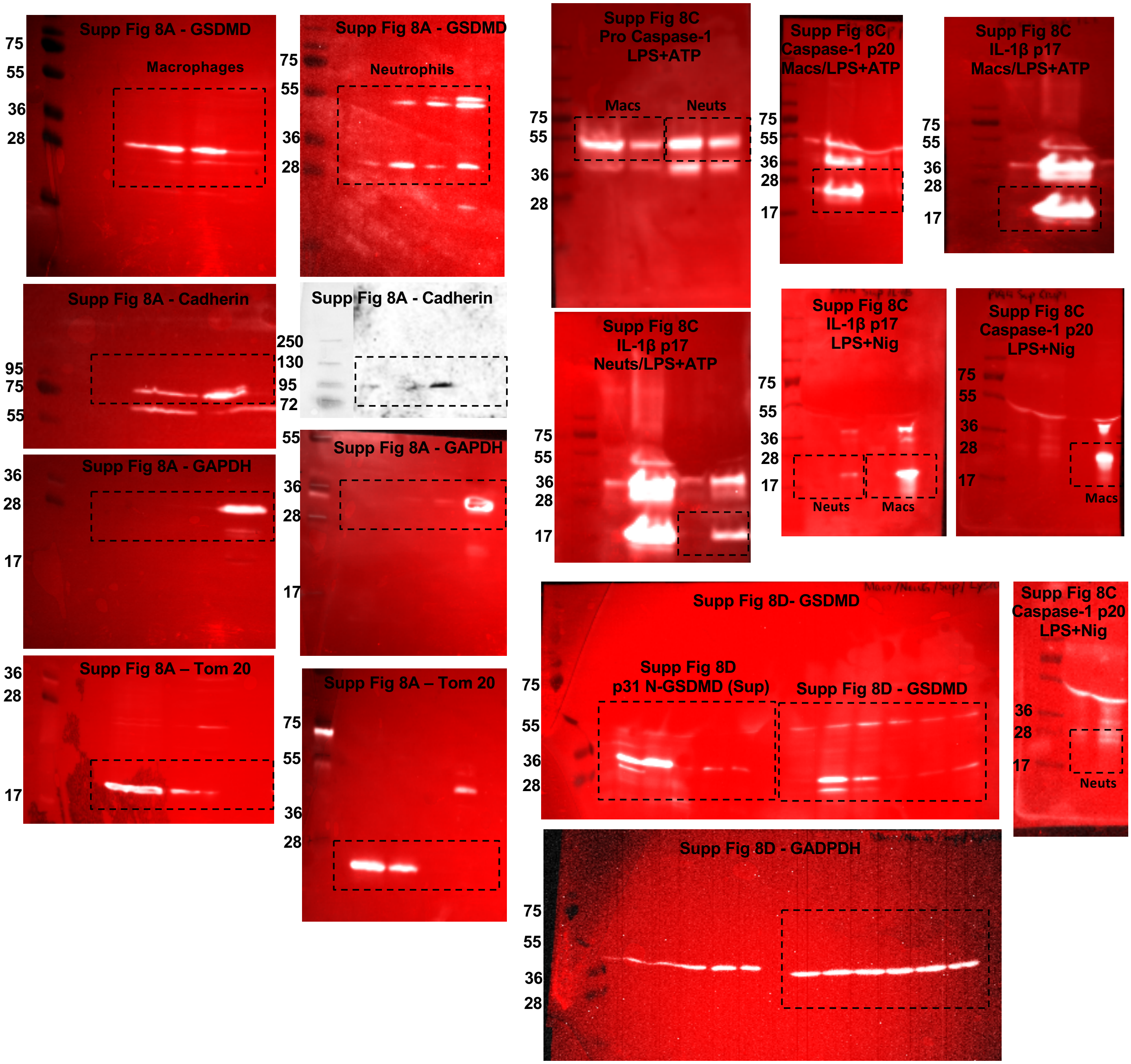

Supplementary Fig 21. ***Un-cropped western blot images for Supplementary Fig 8.***

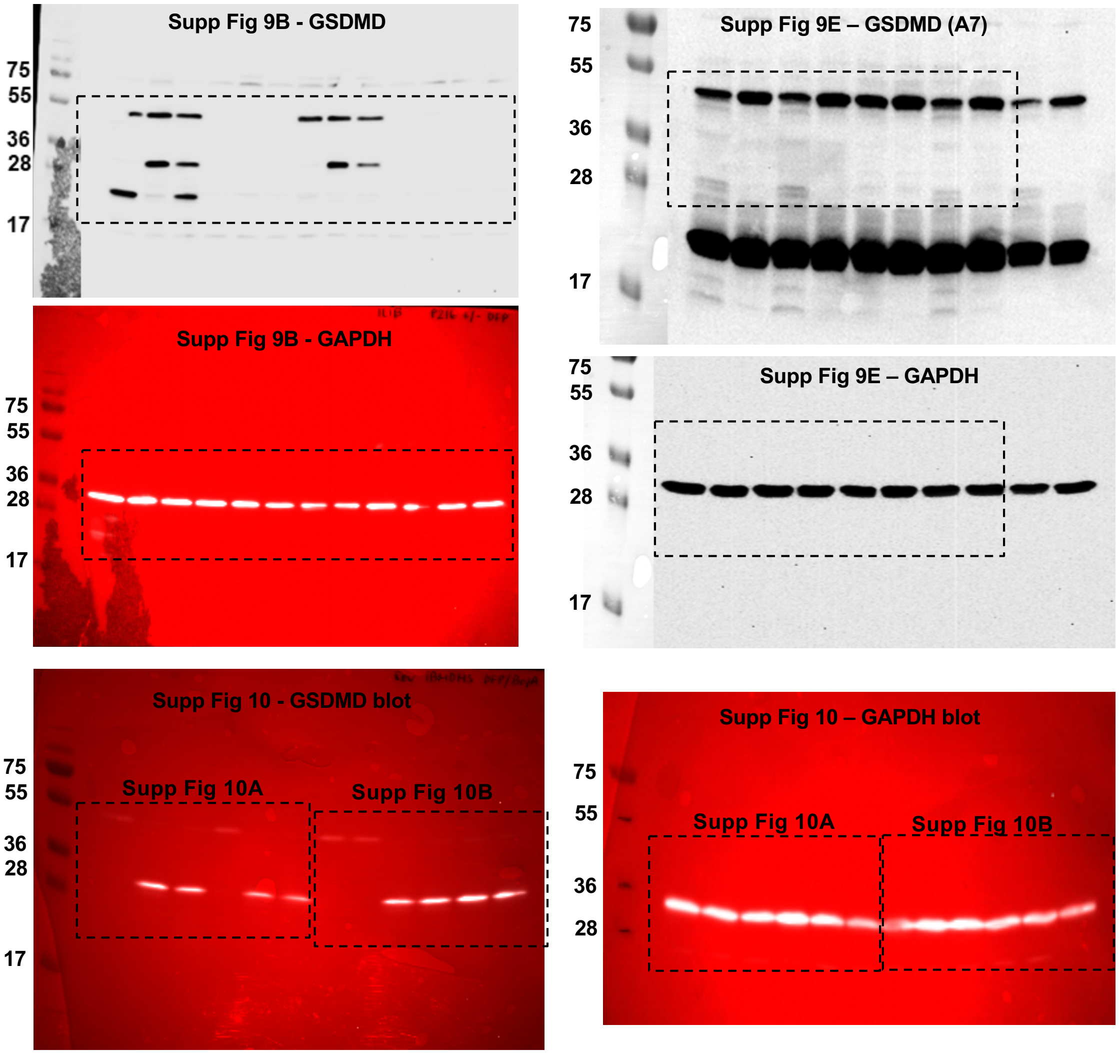

Supplementary Fig 22. *Un-cropped western blot images for Supplementary Fig 9-10.*

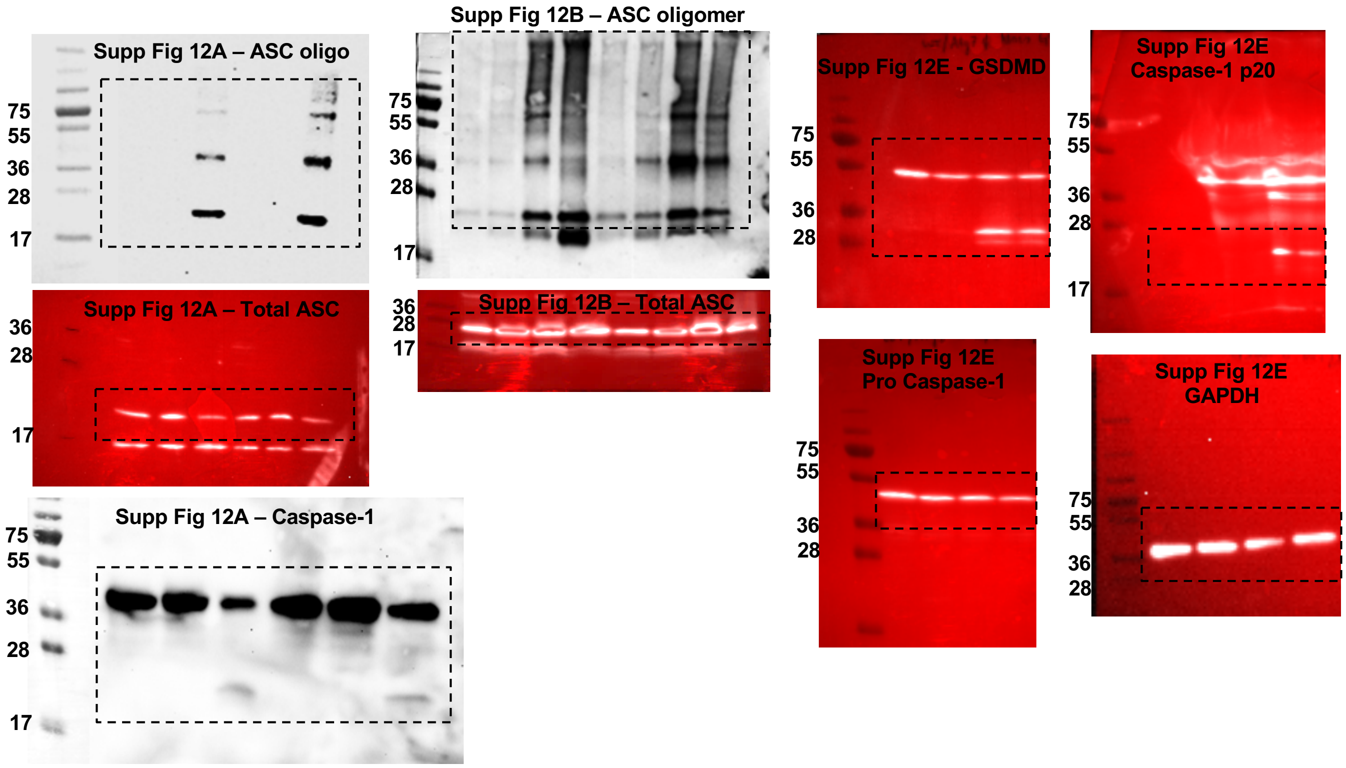

Supplementary Fig 23. ***Un-cropped western blot images for Supplementary Fig 12***
